# Coordinated Activation of Toll-Like Receptor8 (TLR8) and NLRP3 by the TLR8 Agonist, VTX-2337, Ignites Tumoricidal Natural Killer Cell Activity

**DOI:** 10.1371/journal.pone.0148764

**Published:** 2016-02-29

**Authors:** Gregory N. Dietsch, Hailing Lu, Yi Yang, Chihiro Morishima, Laura Q. Chow, Mary L. Disis, Robert M. Hershberg

**Affiliations:** 1 VentiRx Pharmaceuticals, Seattle, WA, United States of America; 2 Tumor Vaccine Group, Center for Translational Medicine in Women’s Health, University of Washington, Seattle, WA, United States of America; 3 Department of Laboratory Medicine, University of Washington, Seattle, WA, United States of America; 4 Department of Medicine, University of Washington, Seattle, WA, United States of America; Exploratory Oncology Research & Clinical Trial Center, National Cancer Center, JAPAN

## Abstract

VTX-2337 (USAN: motolimod) is a selective toll-like receptor 8 (TLR8) agonist, which is in clinical development as an immunotherapy for multiple oncology indications, including squamous cell carcinoma of the head and neck (SCCHN). Activation of TLR8 enhances natural killer cell activation, increases antibody-dependent cell-mediated cytotoxicity, and induces Th1 polarizing cytokines. Here, we show that VTX-2337 stimulates the release of mature IL-1β and IL-18 from monocytic cells through coordinated actions on both TLR8 and the NOD-like receptor pyrin domain containing 3 (NLRP3) inflammasome complex. In vitro, VTX-2337 primed monocytic cells to produce pro-IL-1β, pro-IL-18, and caspase-1, and also activated the NLRP3 inflammasome, thereby mediating the release of mature IL-1β family cytokines. Inhibition of caspase-1 blocked VTX-2337-mediated NLRP3 inflammasome activation, but had little impact on production of other TLR8-induced mediators such as TNFα. IL-18 activated natural killer cells and complemented other stimulatory pathways, including FcγRIII and NKG2D, resulting in IFNγ production and expression of CD107a. NLRP3 activation in vivo was confirmed by a dose-related increase in plasma IL-1β and IL-18 levels in cynomolgus monkeys administered VTX-2337. These results are highly relevant to clinical studies of combination VTX-2337/cetuximab treatment. Cetuximab, a clinically approved, epidermal growth factor receptor-specific monoclonal antibody, activates NK cells through interactions with FcγRIII and facilitates ADCC of tumor cells. Our preliminary findings from a Phase I open-label, dose-escalation, trial that enrolled 13 patients with recurrent or metastatic SCCHN show that patient NK cells become more responsive to stimulation by NKG2D or FcγRIII following VTX-2337 treatment. Together, these results indicate that TLR8 stimulation and inflammasome activation by VTX-2337 can complement FcγRIII engagement and may augment clinical responses in SCCHN patients treated with cetuximab.

**Trial Registration:** ClinicalTrials.gov NCT01334177

## Introduction

Natural killer (NK) cells play an important, well-documented role in cancer immune surveillance and form a bridge to transition innate immune responses to adaptive responses. Activating receptors, such as NKG2D expressed by NK cells, recognize stress-induced ligands on virally infected and malignant cells. Alternatively, NK cell recognition of antibody coated tumor cells through surface FcγRIII/CD16, provides a potent activation signal leading to antibody-dependent cell-mediated cytotoxicity (ADCC), [1, 2]. Both pathways of tumor cell recognition trigger NK cells to secrete cytokines such as IFNγ, and release cytolytic proteins including perforin and granzymes, that induce tumor cell death through the activation of an apoptotic cascades.

ADCC is a well-established effector pathway that contributes to the therapeutic activity of monoclonal antibodies (mAbs) such as cetuximab, an epidermal growth factor receptor (EGFR)-specific mAb approved for treatment of patients with squamous cell carcinoma of the head and neck (SCCHN). VTX-2337 is a selective toll-like receptor 8 (TLR8) agonist that is more potent than either resiquimod (R848) or 3M-002 (CL075) [3], which is currently in Phase 2 clinical development in multiple oncology indications. Treatment of peripheral blood mononuclear cells (PBMC) with VTX-2337 in vitro activates NK cells, enhances trastuzumab-, rituximab- and cetuximab-mediated ADCC, and augments tumor killing through other recognition pathways such as NKG2D [4, 5]. Modulation of NK cell function by TLR8 agonists has important implications for enhancing the therapeutic activity of clinically approved mAbs. Increased ADCC by NK cells may lead to a more vigorous anti-tumor response in the short term, which can help shape tumor-directed adaptive immune responses with the potential for long-term, durable clinical responses [6].

Soluble mediators such as IL-18 are produced by activated macrophages and myeloid dendritic cells (mDC) and enhance NK cell responses invoked by other stimulatory pathways such as Fc receptors and NKG2D [7–8]. TLR ligation and downstream activation of NFkB leads to the synthesis and subsequent accumulation of pro-IL-1β and pro-IL-18 within responsive cells. While this priming step is necessary, the release of mature IL-1 family cytokines is dependent on cleavage of the pro-cytokines by activated caspase-1, which is recruited to the NOD-like receptor pyrin domain containing 3 (NLRP3) inflammasome complex. This second activation signal has generally been linked to perturbations in normal cell physiology, or damage signals, such as uric acid crystals, extracellular ATP, or lysosomal damage, rather than specific ligands [9–10]. Interestingly, TLR8 activation of mDC and monocytes by VTX-2337 in the absence of other activating signals, leads to release of both IL-1β and IL-18 and complements the activities of other mediators induced in response to TLR8 activation [3, 11–13].

In this report, we have elucidated the mechanism of coordinated TLR8 and NLRP3 activation by VTX-2337, which leads to the production and release of IL-18. We have also established that activation of this pathway is not limited to vitro assays, but also occurs in preclinical studies conducted in cynomolgus monkeys. Additionally, we have evaluated how VTX-2337-mediated NLRP3 activation and the downstream production of IL-18 complement canonical pathways of NK activation, such as engagement of NKG2D and FcγRIII receptors. Finally, we describe increased NK cell function in SCCHN patients treated with VTX-2337 in combination with cetuximab. Our results suggest that patient NK cells become more responsive to stimulation by NKG2D or FcγRIII following VTX-2337 treatment. Together, these results indicate that TLR8 stimulation and inflammasome activation by VTX-2337 can complement FcγRIII engagement and may augment clinical responses in SCCHN patients treated with cetuximab.

## Materials and Methods

### Reagents

Fluorochrome-conjugated mAbs against CD3, CD16, CD56, IFNγ, and CD107a were purchased from eBiosciences (San Diego, CA). RPMI culture media, phosphate-buffered saline (PBS), penicillin-streptomycin, and L-glutamine were purchased from Invitrogen Life Technologies (Grand Island, NY). VTX-2337 (VentiRx Pharmaceuticals, Seattle, WA) is a synthetic, small molecule TLR8 agonist based on a 2-aminobenzazepine core structure, previously described and characterized [3]. Acetone was purchased from Fisher Scientific (Houston, TX). The neutralizing anti-IL-18 mAb was purchased from Invitrogen-Biosource (Camarillo, CA).

### Analysis of IL-1β secretion and caspase-1 activation in THP-1 cells

Both wild type THP-1 and NLRP3 defective (NLRP3^def^) THP-1 cells were purchased from InvivoGen (San Diego, CA, Catalog numbers: thp-null and thp-dnlp). THP-1 cells or NLRP3^def^ THP-1 cells (180,000 cells/well) were seeded in a 96-well plate, differentiated with 12-*O*-tetradecanoylphorbol-13-acetate (TPA), and treated with VTX-2337 (3 nM to 10 μM) overnight to assess IL-1β secretion. Levels of IL-1β in culture supernatant were analyzed using an ELISA kit from eBiosciences. In some studies, HEK-Blue^™^ IL-1β cells (InvivoGen), were used to assess levels of IL-1β secreted into the media by THP-1 cells. The production of secreted embryonic alkaline phosphatase (SEAP) by HEK-Blue^™^ IL-1β cells in response to IL-1 was quantified using the QUANTI-Blue detection media and reported as OD650.

### Western blot analysis of IL-1 β, pro-IL-1β, and caspase-1

Protein levels of IL-1β, pro-IL-1β, and caspase-1 in both culture supernatant and whole cell lysate were evaluated using Western blot. After washing, the cells were re-suspended in serum-free RPMI with or without VTX-2337 (3 or 10 μM). Lysates of THP-1 cells were prepared by using the RIPA lysis buffer (0.15M NaCl, 1% NP-40, 0.1% SDS, 50 mM Tris pH8.0) with protease inhibitors. Proteins in culture supernatant from control or VTX-2337 treated THP-1 cells were precipitated using acetone. Proteins were separated on a 12% SDS-PAGE gel (Invitrogen) and transferred to nitrocellulose membrane. The membrane was first probed with anti-IL-1β mAb (clone 3ZD, from NCI-FCRDC), anti-caspase-1 mAb (clone D57A2, from Cell Signaling Technology, Boston, MA), or anti-β-actin (clone 13E5, from Cell Signaling Technology). After washing, the membrane was incubated with horseradish peroxidase-labeled secondary antibodies allowing the signal for IL-1β and caspase-1 to be detected by chemiluminescence (SuperSignal West Pico, Thermo Scientific, Rockford, IL). For β-actin, the membrane was incubated with DyLight-labeled secondary antibody and the signal was detected using LI-COR Odyssey.

### In vitro studies utilizing human PBMC

Astarte Biologics (Redmond, WA) was contracted by VentiRx Pharmaceuticals to provide blood samples from healthy donors. Therefore, the protocol for the collection of blood by Astarte Biologics was approved by their Institutional Review Board (IRB), Schulman and Associates. The blood collection was performed following written informed consent. PBMC were isolated from the blood by Ficoll gradient centrifugation, suspended in RPMI, placed in 96-well plates (200,000 cells/well), treated with serial dilutions of VTX-2337 (0.003 μM-10 μM), and incubated overnight. The culture supernatant was analyzed for levels of IL-1β and IL-18 by ELISA. To demonstrate that caspase-1 activation is required for VTX-2337 induced IL-1β and IL-18 production, the caspase-1 inhibitor z-VAD-FMK (10 μg/ml, Invitrogen) was added to the culture medium 30 minutes before the addition of VTX-2337 (1 μM). The PBMC were incubated overnight, and the levels of IL-1β, IL-18, IFNγ and TNFα were analyzed by ELISA using kits from eBiosciences.

### Real-time RT-PCR analysis of pro-IL-1β and pro-IL-18 mRNA expression

Total RNA was extracted from THP-1 cells or PBMC isolated from blood obtained from healthy donors by Astarte Biologics, as previously described, was treated with VTX-2337 (3 or 10 μM, 24 h) using an RNAqueous4PCR kit (Ambion, Austin, TX). The cDNA was synthesized using the Superscript III reverse transcription kit (Invitrogen). Real-time Taqman PCR was run on an ABI 7900HT instrument using primer and probes from Applied Biosystems (Foster City, CA). The level of target gene expression was normalized to β-actin using the delta Ct method as previously described [14].

### TruCulture^®^ blood collection and whole blood culture system

Whole blood from healthy volunteers was collected using the TruCulture^®^ system (Myriad RBM, Inc., Austin, TX) as previously described [15]. As previously described, Astarte Biologics was contracted to VentiRx Pharmaceuticals to perform the collection. The blood was collected following written informed consent under a protocol approved by the IRB used by Astarte. The TruCulture^®^ syringe tubes used in this study were preloaded with culture medium only, or VTX-2337 at a final concentration of 0.3 or 1 μM and stored at -20°C, then thawed the day before blood collection. Blood was drawn into the tubes and incubated in a dry heat block incubator for 24 h. Following the incubation, cells were separated from the supernatant using the supplier plunger, then frozen at -20°C until the time of cytokine quantification using the human MAP v.1.6 inflammation panel (Myriad RBM).

### Flow cytometric analysis of CD107a and IFNγ expression by NK cells

To evaluate NK cell activation, PBMC were cultured overnight in RPMI + 10% Human AB serum with or without VTX-2337 (0.50 μM). Some samples were further stimulated by exposing the PBMCs to either K562 tumor cells at a 5:1 ratio or to plate-bound anti-CD16 mAb for the last 5 h of incubation. Brefeldin A was included during the last 4 h of incubation to block the secretion of IFNγ, while CD107a-PE was added for 5 h. Following the culture activation, cells were stained with fluorophore-conjugated antibodies to surface markers including anti-CD3-Alexa 488, anti-CD16-PerCP-Cy5.5, and anti-CD56-APC. After subsequent fixation and permeabilization, the cells were stained with anti-IFNγ-eFluor450. Samples were analyzed using a FACS Canto II instrument, and data collected in list mode were analyzed using Flow Jo software (Ashland, OR).

### *In vivo* administration of VTX-2337 in cynomolgus monkeys

Studies in cynomolgus monkeys were conducted at Charles River Laboratories (CRL), Preclinical Services, (Shrewsbury MA) in strict accordance with the recommendations in the Guide for the Care and Use of Laboratory Animals of the National Institutes of Health. The study was reviewed and approved by the CRL Institutional Animal Care and Use Committee, under submission number DPKW-101. Study animals were colony animals that were returned to the colony on completion of the study. The male monkeys (2.9–4.9 kg) were housed individually (cage dimensions of 0.76 m wide x 0.74 m deep x 0.81 m in height), but commingled periodically as part of the environmental enrichment program. The animals were also given fruit, vegetable, or additional supplements as a form of environmental enrichment, as well as given various cage enrichment devices. Animals were given Certified Primate Diet #2055C (Harlan Teklad), two times daily and water ad libitum. Environmental controls for the housing were set to maintain 18–26°C, a relative humidity of 30–70%, a minimum of 10 room air changes/h and a 12-h light/12 h dark cycle. While doses of VTX-2337 were well tolerated, provisions including use of anti-inflammatory agents to moderate the immune response were considered in the study design.

VTX-2337 was administered as a bolus subcutaneous (SC) injection in the intrascapular area at doses of 1 and 10 mg/kg. Blood samples were collected at baseline (pre-dose), and 6, 12, 24, and 96 h post injection to monitor levels of IL-1β and IL-18 in the plasma using the human MAP v.1.6 inflammation panel (Myriad RBM). Due to the routine, non-invasive procedures for dosing and blood collection, anesthetics were not considered necessary for the study.

### Administration of VTX-2337 to patients with head and neck cancer and immune monitoring of NK cell responses in treated patients

The safety and tolerability of cetuximab in combination with VTX-2337 was evaluated in a Phase 1 clinical trial in adult patients with advanced recurrent squamous cell carcinomas of the head and neck (SCCHN) (Study A103; ClinicalTrials.gov NCT01334177). The study was conducted at a single study center (University of Washington, Seattle Cancer Care Alliance, Seattle, WA, USA) from June 2011 to June 2014 and was performed in accordance with good clinical practice guidelines and the ethical principles outlined in the Declaration of Helsinki. Approval for study procedures was obtained from the institutional review board of the study site, and all subjects provided written informed consent before study enrollment. Patients who were eligible for this study were adults with advanced or recurrent SCCHN that was no longer amenable to treatment by surgery or radiation therapy or patients with distant or metastatic disease.

The primary objective this study was to determine the safety, tolerability and to assess the principal toxicities of VTX-2337 when given in conjunction with cetuximab. The secondary objective was to determine the pharmacodynamic response of VTX-2337 in combination with cetuximab. The primary endpoint was to determine the maximum tolerated dose (MTD)/recommended Phase 2 dose (RP2D) and to define the toxicities of VTX-2337 in combination with cetuximab. Secondary endpoints included the analysis of biologic correlative assays. The sample size was depended upon the observed safety profile, which determined the number of patients per dose level and the number of dose escalations.

**Fig 1:**
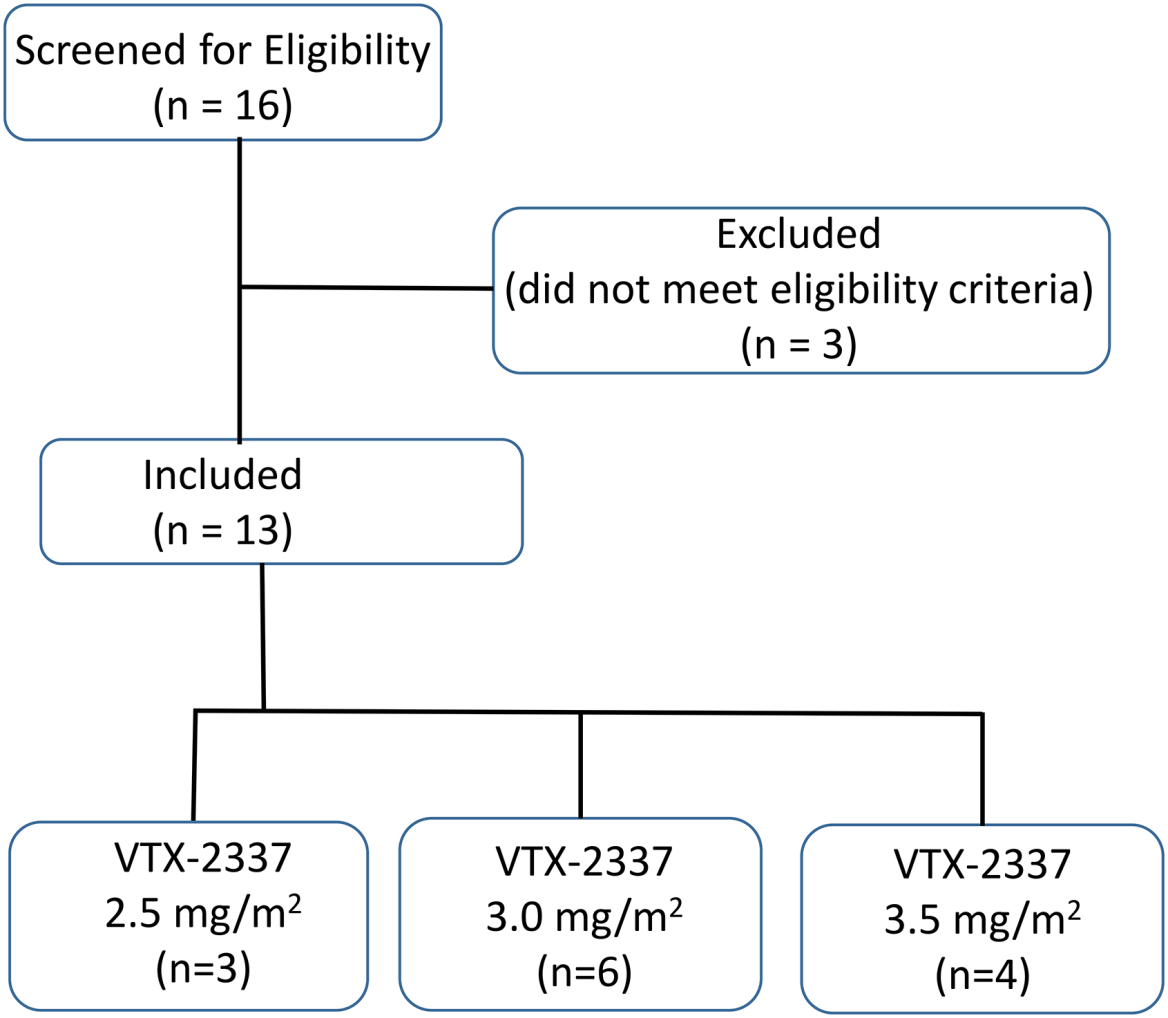
CONSORT Flow Diagram for Phase 1 clinical study in adult patients with advanced recurrent squamous cell carcinomas of the head and neck (SCCHN). Study subjects received VTX-2337 administered by the SC route on days 1, 8 and 15 of a 28-day treatment cycle. The first cohort received a 2.5 mg/m^2^ dose of VTX-2337 following cetuximab administration; this dose was escalated in subsequent cohorts using a 3+3 design to 3.0 mg/m^2^ and finally 3.5 mg/m^2^.

Study medications (cetuximab and VTX-2337) were administered in the clinic by appropriately qualified and trained personnel. This was an open-label study with no blinding. Each patient in this dose-escalation study was assigned to a dose level of VTX-2337 at the time of study enrollment. For each cohort, cetuximab was administered using a loading dose (400 mg/m^2^ IV), followed by a weekly maintenance dose (250 mg/m^2^, IV). Each cetuximab dose was administered as an IV infusion: the initial dose was infused over 2 h, subsequent doses were administered over 1 h. VTX-2337 was administered by the SC route on days 1, 8 and 15 of a 28-day treatment cycle. The first cohort received a 2.5 mg/m^2^ dose of VTX-2337 following cetuximab administration; this dose was escalated in subsequent cohorts using a 3+3 design to 3.0 mg/m^2^ and finally 3.5 mg/m^2^. After successful completion of Cycle 1, patients were eligible to receive subsequent treatment cycles until the criteria for study discontinuation or withdrawal were met, including disease progression, intolerable toxicity, or death. A Consort Flow Diagram for the Clinical study evaluating VTX-2337 in adults with advanced or recurrent SCCHN is provided as Fig 1. The Study Protocol, VTX-2337 Phase 1 Trial in SCCHN Protocol A103 is available as supporting information, S1 Protocol. A TREND Statement Checklist for the study is provided as supporting information, S1 Checklist.

Heparinized blood was collected pre-VTX-2337 dose and at 24 h post-dose to assess NK cell function. Freshly isolated PBMC were cultured in medium alone, stimulated with K562 cells that express transmembrane IL-15 and the 4-1-BB ligand (K562-15mb-41BBL) (16) at a 5:1 ratio, or stimulated with plate-bound anti-CD16 mAb (3G8, BD Biosciences, San Jose, CA). Flow cytometric measurement of surface CD107a expression in CD3^-^CD56^+^ NK cells was performed similarly to the method described above, using anti-CD3-AF488, anti-CD56-APC, and anti-CD107a-PE (all, eBioscience, San Diego, CA).

### Statistical analysis

Statistical analysis was performed using GraphPad Prism, version 6 for Windows software (San Diego, CA). Differences between the treatment groups were analyzed using the two-tailed unpaired Student’s t test or the Wilcoxon signed-rank test for matched pair analysis when normal distribution could not be assumed. A value of p<0.05 was considered statistically significant. Missing data was not imputed.

**Fig 2:**
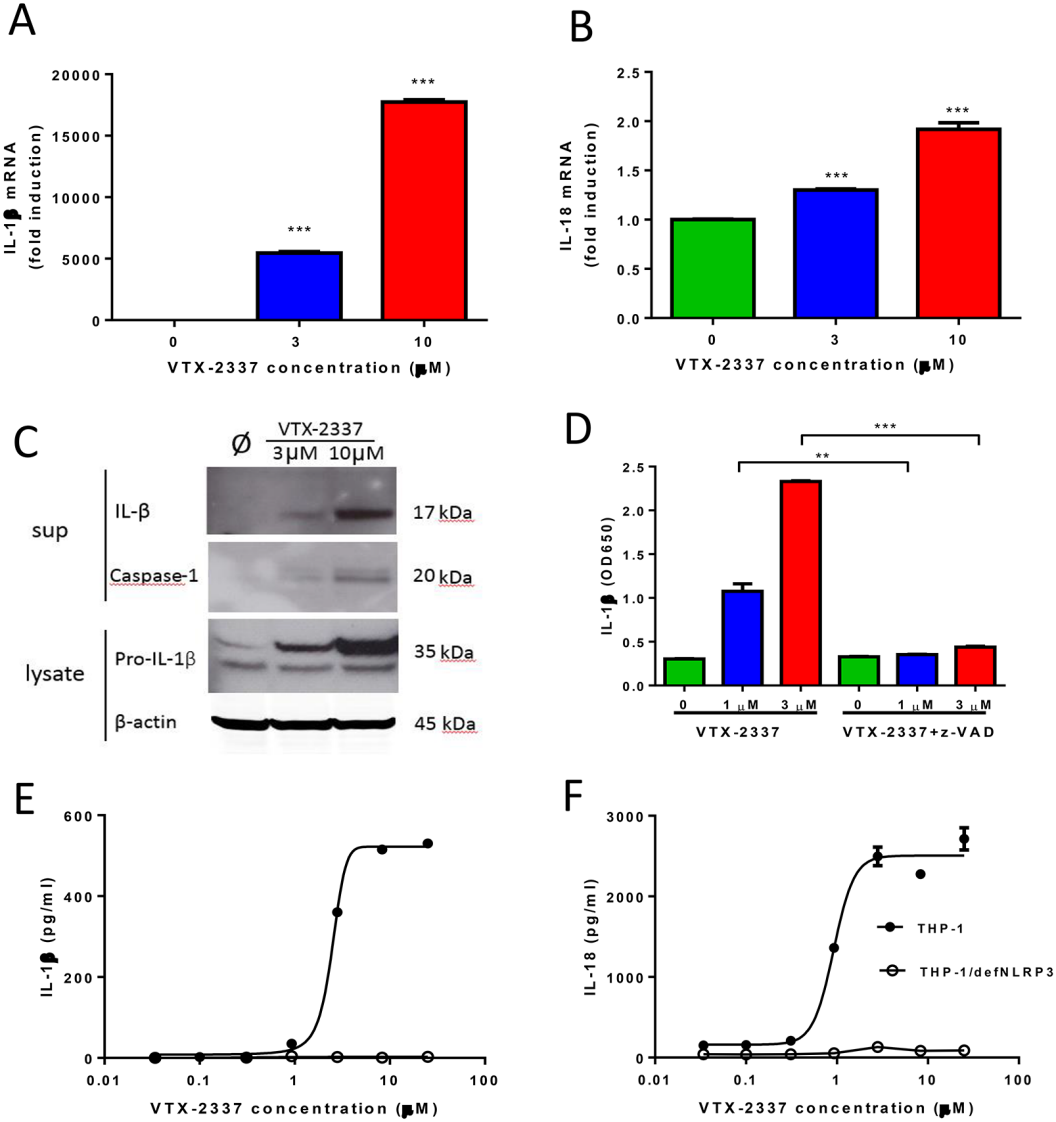
VTX-2337 induces mature IL-1β and IL-18 via NLRP3 inflammasome activation in THP-1 cells. (A-B) VTX-2337 induced pro-IL-1β and pro-IL-18 mRNA expression in THP-1 cells. Cells were cultured overnight in medium or VTX-2337 (3 or 10 μM), and expression of IL-1β and IL-18 mRNA, normalized to β-actin levels, were measured by real-time RT-PCR. (C) Western blot analysis of IL-1β and caspase-1 protein levels in THP-1 culture supernatants, and pro-IL-1β protein levels in cell lysates, following overnight activation with medium (Ø), or VTX-2337 (3 or 10 μM). (D) Pretreatment with caspase-1 inhibitor z-VAD blocks VTX-2337-induced secretion of mature IL-1β. Triplicate THP-1 cultures (10 μg/ml) were treated with VTX-2337. Levels of IL-1β in the presence or absence of z-VAD-FMK were assessed using HEK-Blue^™^ IL-1β cells (mean±sem). (E and F) Levels of secreted IL-1β and IL-18 following overnight VTX-2337 stimulation (0.03 to 25 μM in 1:3 serial dilutions) in wild type THP-1 (●) and NLRP3 deficient THP-1 (THP-1/NLRP3^def^) cells (○). Levels of IL-1β and IL-18 (mean±sem) in culture supernatant from triplicate wells were measured by ELISA. All results shown are representative of three independent experiments. **, *p*<0.01, ***, *p*<0.001 by unpaired Student t-test.

**Fig 3:**
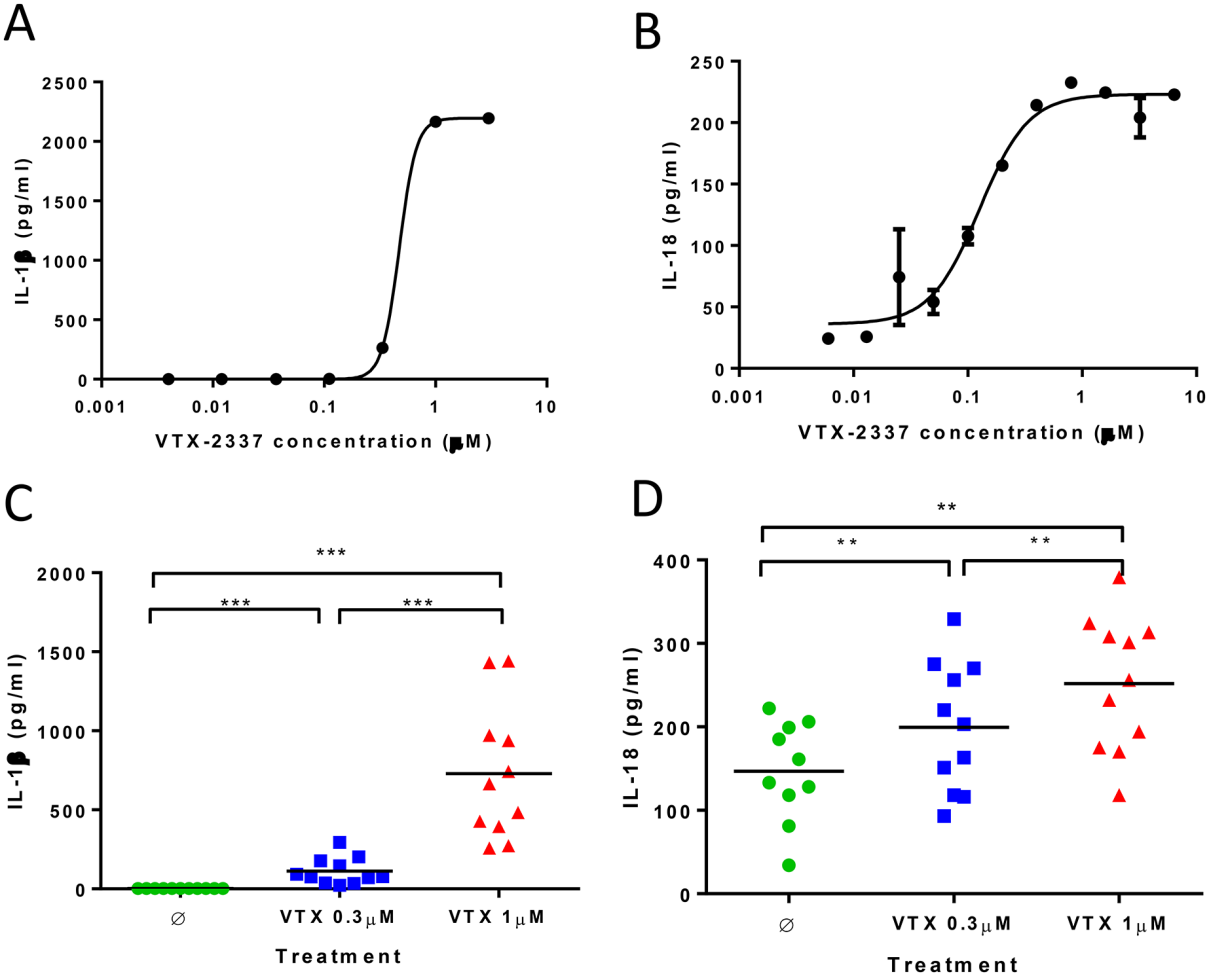
VTX-2337 induces IL-1β and IL-18 in human PBMC. (A-B) IL-1β and IL-18 secretion from VTX-2337 activated PBMC. Dose responses are shown for one representative donor, where data point represents the mean for duplicate culture wells at the indicated concentration of VTX-2337 after 24 h. (C-D) Summary graphs of VTX-2337-induced IL-1β and IL-18 in whole blood from 11 donors, as analyzed by TruCulture. Whole blood was incubated with medium (Ø) or VTX-2337 (0.3 or 1 μM) for 24 h, levels of secreted IL-1β and IL-18 were analyzed in a multiplexed assay. Each symbol represents data from an individual donor. The horizontal bar represents group average. **, *p*<0.01; ***, *p*<0.001 by Wilcoxon signed-rank test.

**Fig 4:**
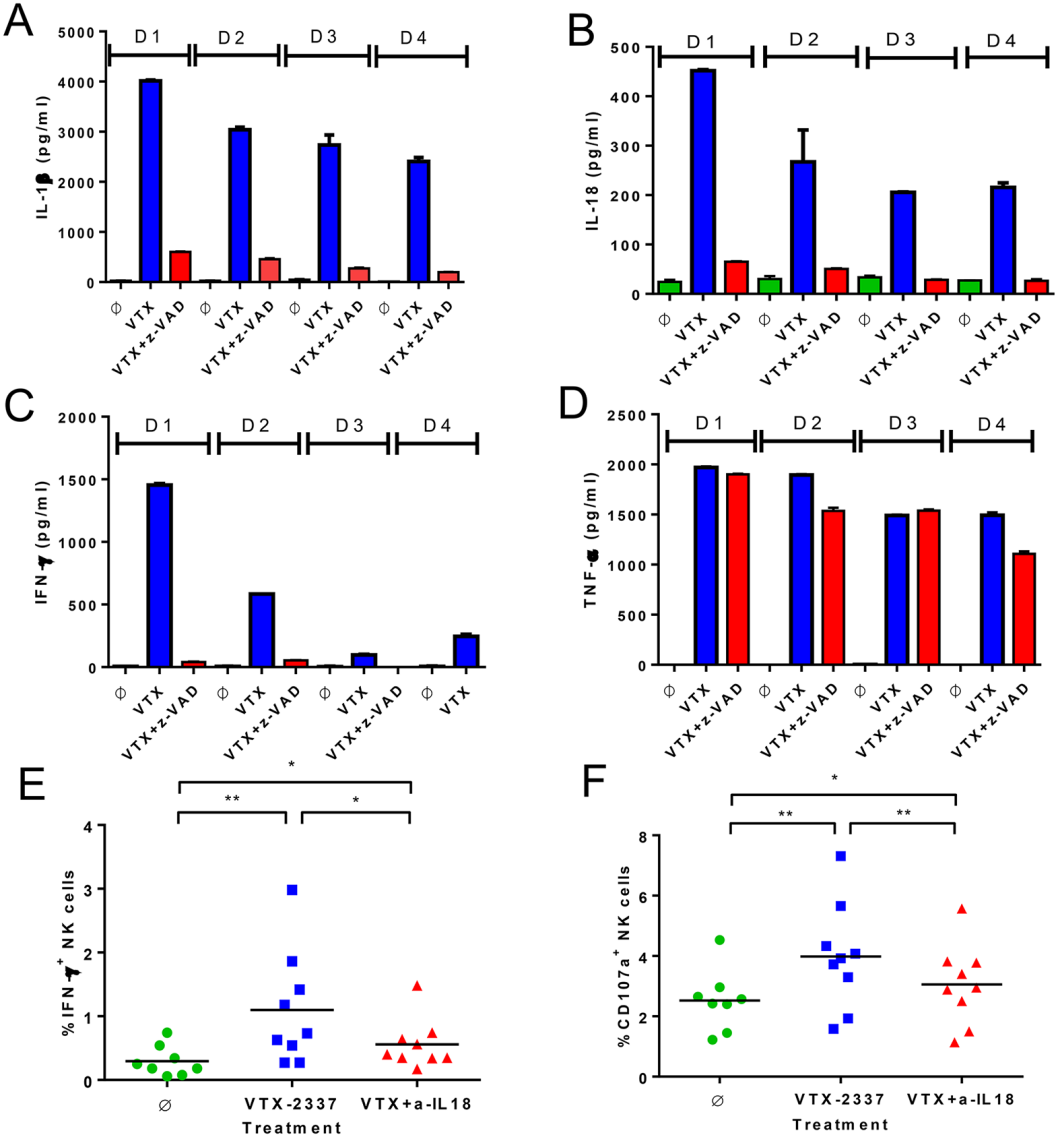
Caspase-1 activation and IL-18 induction by VTX-2337 leads to NK cell activation. (A-D) The effects of caspase-1 inhibition on IL-1β, IL-18, IFNγ, and TNFα production by PBMC. Duplicate PBMC cultures from four healthy donors (indicated as D1, D2, D3 and D4) were treated overnight with medium (Ø), VTX-2337 (1 μM), or VTX-2337 plus z-VAD-FMK (10 μg/ml). Levels of IL-1β, IL-18, IFNγ, and TNFα in culture supernatant were measured by ELISA. (E-F) The effects of IL-18 blockade on VTX-2337-induced IFNγ and CD107a expression in NK cells. Summary graphs show the percentage of IFNγ^+^ and CD107a^+^ NK cells from PBMC cultured with medium (Ø), VTX-2337 (0.5 μM), or VTX-2337 and anti-IL18 neutralizing antibody (20 μg/ml) for 9 donors where each symbol represents one individual donor. The horizontal bar represents group the mean for the treatment. Data shown are the summary of four independent experiments from the 9 donors, *, *p*<0.05 by Wilcoxon signed-rank test.

**Fig 5:**
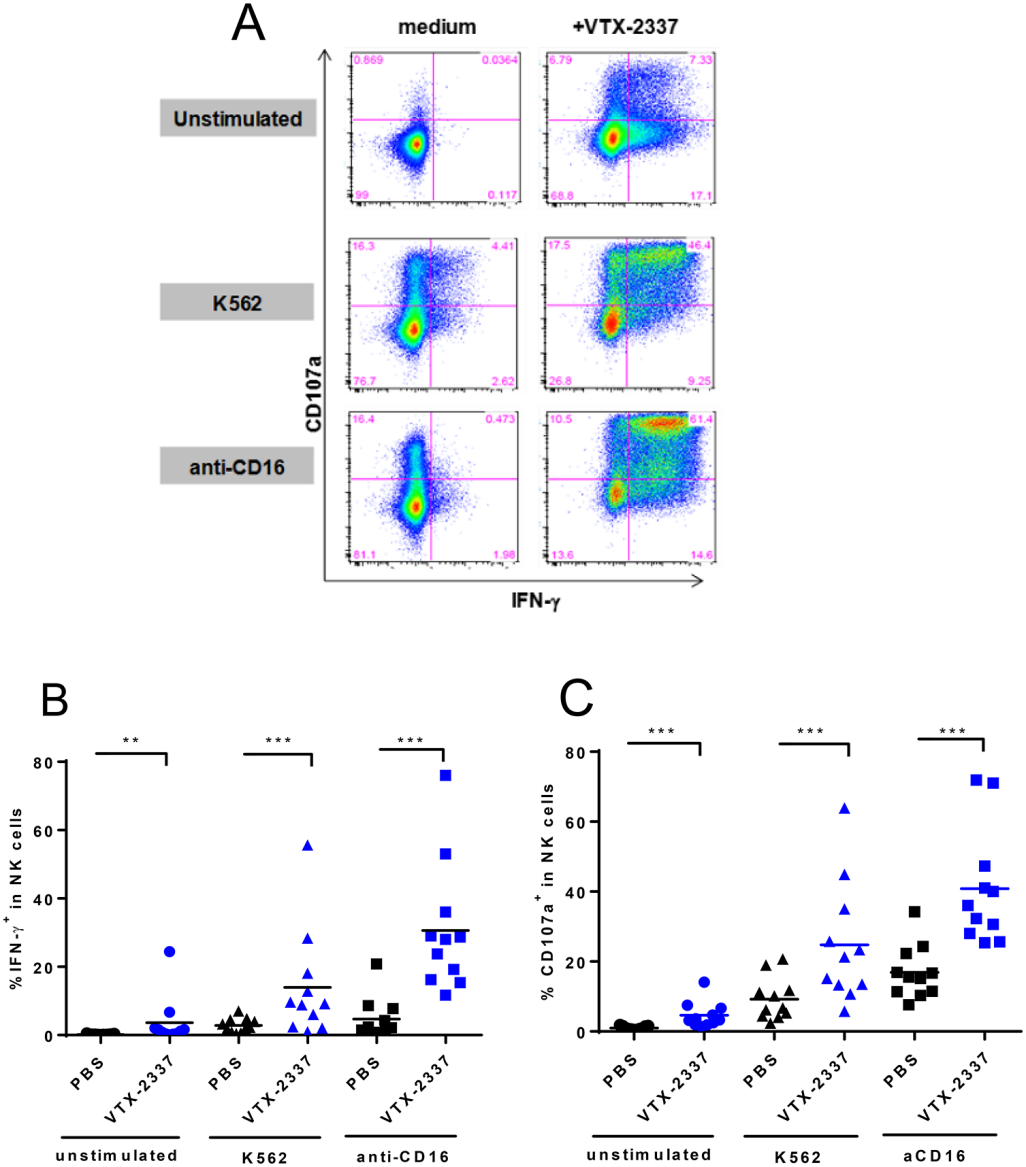
VTX-2337 enhances NK activation in response to stimulation by K562 cells and anti-CD16 *in vitro*. (A) FACS plots showing IFNγ and CD107a expression by NK cells following overnight cultured in medium or VTX-2337 (0.5 μM), and then left unstimulated, stimulated with either K562 cells, or plate-bound anti-CD16 mAb. (B-C) Summary graphs of FACS analyses of NK cell activation in PBMC from 11 donors. Each data point represents an individual donor. The horizontal bar represents group average. Each donor was analyzed in an independent experiment. *, *p*<0.05, **, *p*<0.01, ***, *p*<0.001 by Wilcoxon signed-rank test.

**Fig 6:**
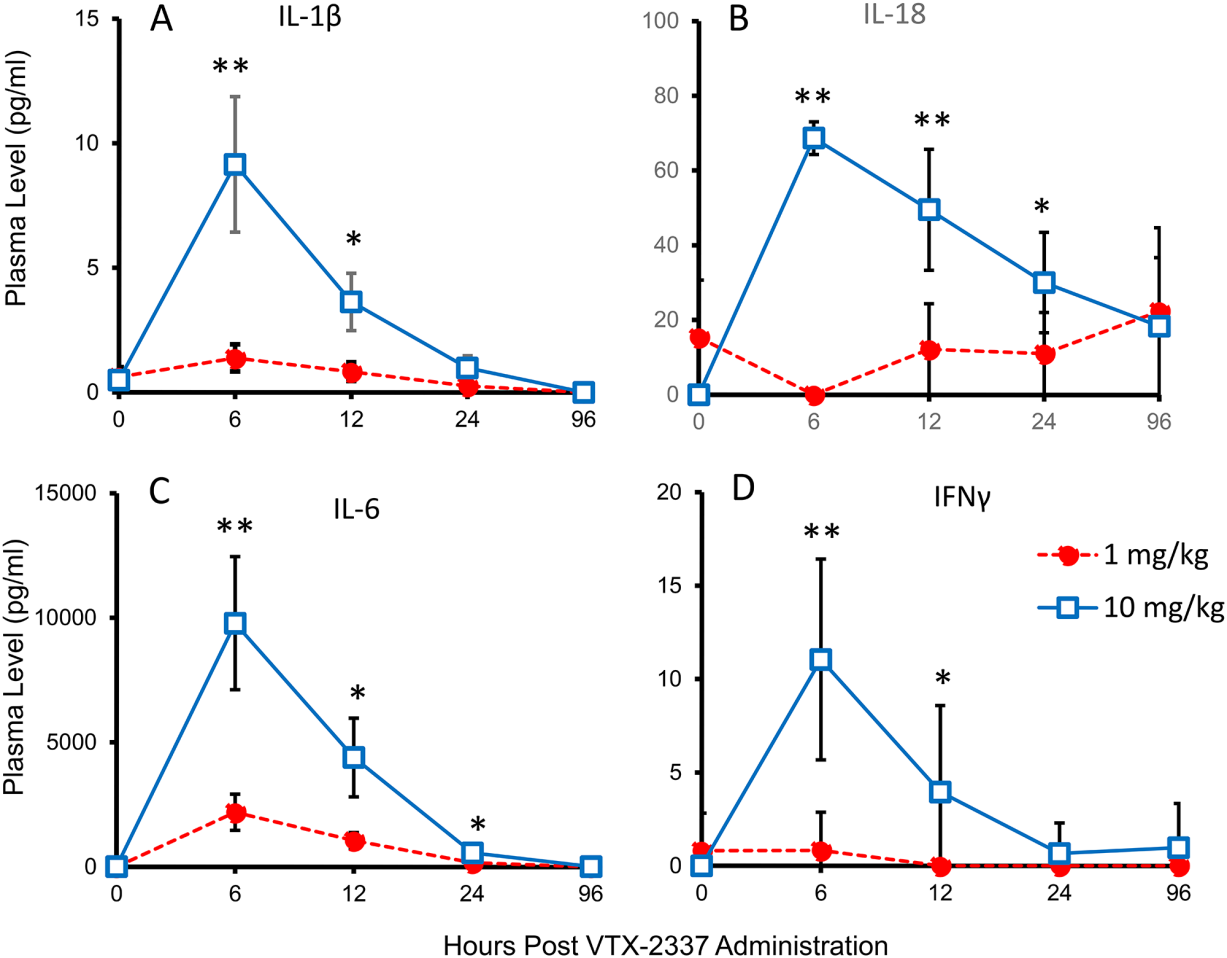
VTX-2337 administration to cynomolgus monkeys induces IL-1β and IL-18. Male cynomolgus monkeys (n = 6 per group) received a subcutaneously injection of VTX-2337 (1 or 10 mg/kg). Plasma collected predose, and 6, 12, 24 and 96 h following dosing was assessed for levels of IL-1β, IL-18 and IL-6. Cytokine levels in pg/mL (mean±sem) at each time point for the two VTX-2337 dose levels are shown. At each VTX-2337 dose level, plasma cytokine levels at 6, 12, 24 and 96 h were compared to predose levels *, *p*<0.05, **, *p*<0.01, by t-test.

The datasets from experiments and studies presented in Figs 2–6 are provided as supporting information: S1 File.

## Results

### VTX-2337 induces NLRP3 inflammasome activation and production of IL-1β and IL-18 by THP-1 cells and PBMC

Treatment of THP-1 cells with VTX-2337 induced a highly significant (p<0.001, unpaired t test), concentration-dependent increase in IL-1β and IL-18 mRNA, consistent with TLR-mediated activation of NF-ĸB (Fig 2A and 2B). Western blot analysis showed induction of both pro-IL-1β (35 kDa) in THP-1 cell lysate and the release of mature IL-1β (17 kDa) into the culture supernatant (Fig 2C). The active form of caspase-1 (p20, MW of 20kD), which is responsible for the cleavage of pro-IL-1β, was also induced in these cells by VTX-2337. Inhibition of caspase-1 with z-VAD-FMK resulted in a marked reduction of IL-1β release from VTX-2337-treated THP-1 cells (Fig 2D). To demonstrate that activation of the NLRP3 inflammasome is required for the generation of mature IL-1β and IL-18 by THP-1 cells, the VTX-2337 response was evaluated in NLRP3-deficient cells. As shown in Fig 2E and 2F, the concentration-related release of mature IL-1β and IL-18 in response to VTX-2337 (0.03–25 μM), did not occur in NLRP3 deficient THP-1 cells.

To look for comparable activity in primary human cells activated with VTX-2337, the induction of IL-1β and IL-18 was assessed using PBMC from healthy donors. In the absence of known activators of NLRP3, VTX-2337 induced the secretion of both IL-1β and IL-18 in a concentration-dependent manner (Fig 3A and 3B). As additional confirmation of this response, whole blood collected from multiple healthy donors (n = 11) was activated using multiple VTX-2337 concentrations, and production of IL-1β and IL-18 was assessed using the MAP1.6 inflammation panel to complement the ELISA results described above. As shown in Fig 3C and 3D), IL-1β levels increased from a mean of 2.6 ± 0.3 ng/mL in untreated controls to 729 ± 424 ng/mL (p < 0.001, Wilcoxon test) for cells activated with 1 μM VTX-2337. There was also a significant, (p<0.01 Wilcoxon test), concentration-dependent increase in IL-18 release in response to VTX-2337 activation.

### Caspase-1 activation and IL-18 production drive NK cell activation by VTX-2337

Caspase-1 activation is an essential step in the processing and secretion of the mature forms of IL-1β and IL-18 [1]. To demonstrate that caspase-1 is activated in response to VTX-2337, PBMC from multiple healthy donors were pretreated with the caspase-1 inhibitor, z-VAD-FMK (10 μg/ml), prior to activation with VTX-2337 (1 μM, 24 h). Pretreatment with z-VAD-FMK reduced IL-1β and IL-18 release from VTX-2337-activated PBMCs by 85–90% (Fig 4A and 4B). Interestingly, z-VAD-FMK also blocked the production of IFNγ, but had little effect on the TNFα response induced by VTX-2337 (Fig 4C and 4D). We have previously reported that NK cells are a major source of IFNγ in VTX-2337-stimulated PBMC [3]. The reduction in IFNγ seen with caspase-1 inhibition is consistent with VTX-2337 driving mDC and monocytes to produce IL-18 that subsequently contributes to NK cell activation. To further evaluate the activity of IL-18 on NK cells, expression of intracellular IFNγ and CD107a, markers of activation and degranulation respectively [17], were monitored by flow cytometry. The number of IFNγ^+^ NK cells increased from 0.30 ± 0.24 in untreated cultures to 1.17 ± 0.91 (p<0.01, Wilcoxon test) with VTX-2337 treatment (Fig 4E). The addition of a neutralizing anti-IL-18 mAb (20 μg/ml) resulted in a partial, but significant reduction in the percentage of NK cells activated by VTX-2337 (p<0.05, Wilcoxon test). Expression of the degranulation marker CD107a by NK cells showed a similar pattern, where VTX-2337 induced a significant increase in expression that was partially blocked by the anti-IL-18 mAb (Fig 4F). These results are consistent with our previous report demonstrating that NK cells respond directly to TLR8 agonists and IL-18 release from activated accessory cells can augment their activation [3].

### VTX-2337 enhances NK cell responses to K562 target cells and FcγRIII (CD16) stimulation *in vitro*

Previous studies have shown that IL-18 provides a stimulatory signal that works cooperatively with the engagement of activating surface receptors to enhance NK cell activation. Based on the release of both IL-12 and IL-18 from TLR8-activated mDC/monocytes, we hypothesized that VTX-2337 should augment NK cell activation in response to K562 target cells that express NKG2D ligands and signaling through FcγRIII cross-linking with anti-CD16 mAbs. To test this hypothesis, PBMC were initially pretreated with VTX-2337 (0.5 μM) for 24 h. NK cells were subsequently stimulated with either K562 tumor cells to activate the NKG2D pathway or plate-bound anti-CD16 mAb to signal through FcγRIII. In the absence of any stimuli, the percentage of IFNγ^+^, CD107a^+^ NK cells was < 0.1% (Fig 5A). As single activation agents, VTX-2337, K562 cells, and anti-CD16 each produced a modest degree of activation, with the percentage of IFNγ^+^, CD107a^+^ NK cells increasing to 7.33%, 4.41% and 0.47%, respectively (Fig 5A). When NK cells were pre-treated with VTX-2337, then exposed to either K562 target cells or immobilized anti-CD16 mAb, there was a much greater effect than with any single stimuli (Fig 5A). Sequential activation with VTX-2337 followed by co-culture with K562 cells increased the population of IFNγ+.CD107a + NK cells to 46.4%, while activation by VTX-2337 followed by FcγRIII stimulation with immobilized anti-CD16 mAb increased this population to 61.4%.

NK cell activation, where VTX-2337 pretreatment was followed by exposure to K562 cells or immobilized anti-CD16 was expanded to assess the response in 11 healthy volunteers. As shown in Fig 5B and 5C, VTX-2337 alone resulted in significant increases in IFNγ+ (p<0.01 Wilcoxon test) and CD107a+ (p<0.001 Wilcoxon test) NK cell populations. The percentage of activated NK cells was markedly augmented when VTX-2337 pretreatment was followed by co-culture with K562 cells or immobilized anti-CD16 treatment. The VTX-2337-mediated activation of NK cell subsets, CD56^bright^ and CD56^dim^ cells, was also assessed (S1 Fig). Both NK cell subsets were activated by VTX-2337 as demonstrated by increased IFNγ and CD107a expression, and the response was enhanced by stimulation of NKG2D by K562 target cells or FcγRIII using immobilized anti-CD16 mAb.

### *In vivo* administration of VTX-2337 in cynomolgus monkeys induces circulating IL-1β and IL-18

To demonstrate that VTX-2337 drives the production of both pro-IL1β and pro-IL18, and activates the NLRP3 inflammasome in the absence of any other activating signals, cynomolgus monkeys were treated with the compound and plasma was monitored for these mediators. Cynomolgus monkey were chosen as they are highly responsive to VTX-2337 and predictive of the human TLR8-mediated response. This is in contrast to rodent species, where agonists optimized for activity on human TLR8 have limited activity due to sequence differences in the molecules ecodomain [18].

Monkeys received a subcutaneous injection of VTX-2337 (1 or 10 mg/kg), and plasma was collected predose, 6, 12, 24, and 96 h post-injection. For the 10 mg/kg dose, mean plasma levels of IL-1β increased from baseline levels of 0.5 pg/mL, up to 9.12 ± 2.7 ng/mL (p<0.05, t-test) at 6 h post-administration of VTX-2337 (10 mg/kg, Fig 6A). Circulating levels of IL-18 also increased from a baseline of ~ 1 pg/mL to 68.7 ± 4.4 pg/mL (p < 0.05, t-test) at 6 h in response to the VTX-2337 treatment (10 mg/kg, Fig 6B). Levels of IL-6 were monitored (Fig 6C), as this mediator is induced in response to TLR8 activation, but the release is independent of NLRP3 inflammasome activation. In addition, plasma levels of IFNγ were assessed as a measure of NK cell activation in response to VTX-2337 treatment (Fig 6D). As expected, this biomarker was undetectable in plasma prior to treatment, and increased to 11.1 ± 5.4 pg/mL at 6 h in monkeys administered the 10 mg/kg dose of VTX-2337. Overall, these results demonstrate that the coordinated activation of TLR8 and NLRP3 by VTX-2337 observed in vitro studies also occurs in vivo. Additionally, the relatively low levels of plasma IL-1β and IL-18 relative to IL-6 is consistent with the hypothesis that production of IL-1 family cytokines is more tightly regulated to minimize collateral damage to the host [19].

### Treatment of SCCHN patients with VTX-2337 enhances NK cell activation

Thirteen patients with recurrent or metastatic SCCHN were enrolled on Study A103. Cetuximab was administered in combination with 3 escalating dose levels of VTX-2337: 2.5 mg/m^2^ (n = 3), 3.0 mg/m^2^ (n = 6), and 3.5 mg/m^2^ (n = 4). The median age was 62 years (range, 51 to 78) and the majority of patients (10 of 13; 77%) were male. The study population was generally representative of patients with recurrent or metastatic SCCHN. A Consort Flow Diagram for the study is shown in Fig 1.

NK cell activation was assessed in two SCCHN patients treated with 3.0 mg/m^2^ VTX-2337. Blood samples were collected prior to VTX-2337 treatment and again 24 h following VTX-2337 administration. Isolated PBMC from the pre- and post-VTX-2337 treatment blood samples were placed into culture for 4 h without additional stimulation or with *ex vivo* stimulation using either K562-15mb-41BBL target cells [16], or immobilized anti-CD16 mAb, as previously described. Activation of the NK cell population was monitored by increased expression of CD107a.

**Fig 7:**
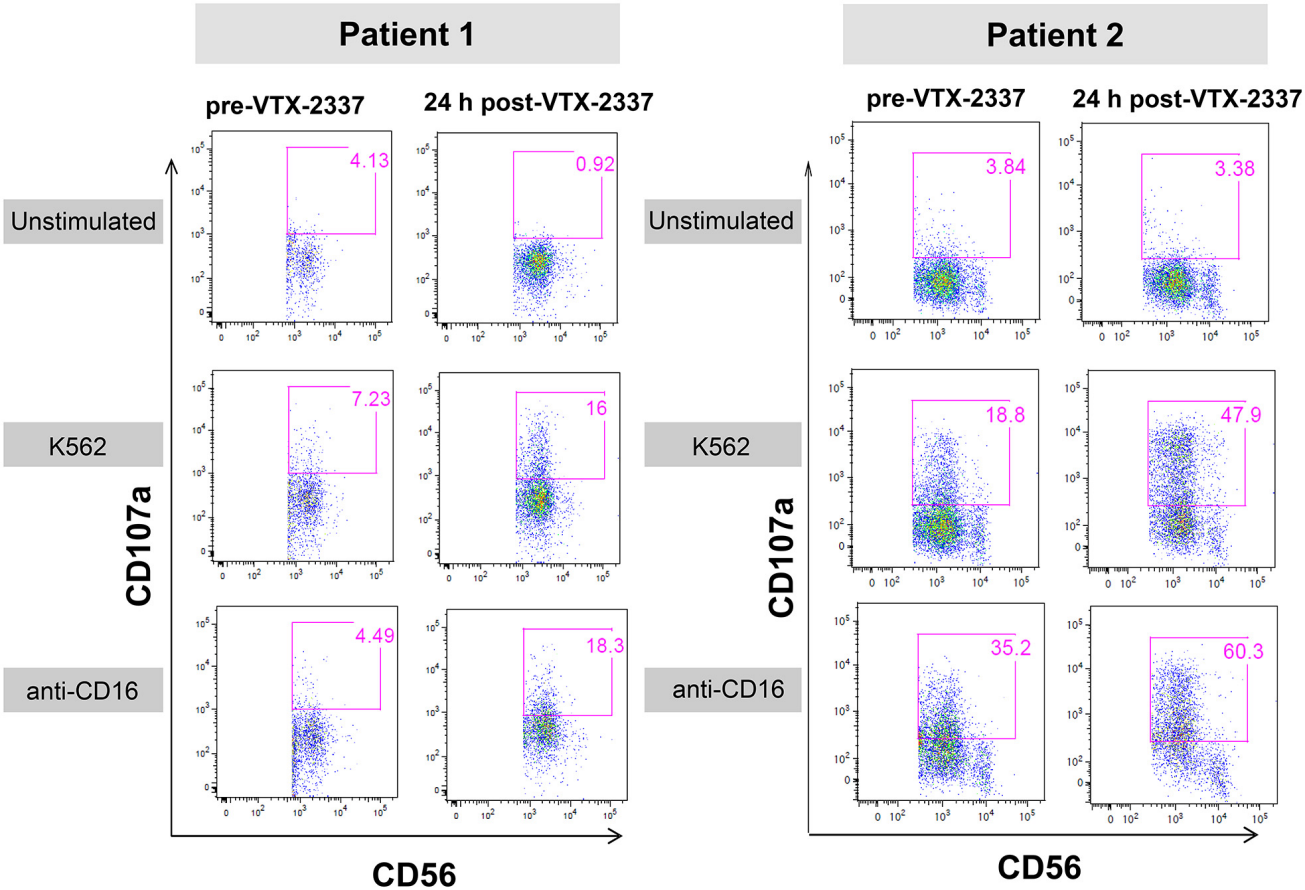
Administration of VTX-2337 to SCCHN cancer patients enhances NK cell degranulation. Representative FACS plots from two patients illustrate NK cell activation measured by increased CD107a expression 24 h following the administration of VTX-2337 (3.0 mg/m^2^) compared to pre-treatment. Freshly isolated PBMC from each patient were unstimulated (negative control), or were stimulated with K562-15mb-41BBL cells or plate-bound anti-CD16 mAb.

In blood samples collected prior to VTX-2337 administration, the prevalence of CD107a-expressing CD56+ NK cells in the control cultures was low (3.8–4.1%) for both patients, as shown in Fig 7. For Patient 1, the *ex vivo* stimulation with either the K562 cells or immobilized anti-CD16 mAb, produced only a small increase in the percent of CD107a+ CD56+ NK cells. In contrast, *ex vivo* stimulation of PBMC from Patient 2 with the K562 cells increased the percent of CD107a+ CD56+ NK cells to 18.8%, while the immobilized anti-CD16 mAb increase the percentage to 35.2%.

Following VTX-2337 treatment, the percent of CD107a-expressing CD56+ NK cells in the unstimulated control cultures remained low for both patients. However, the introduction of a second activation signal by *ex vivo* stimulation with either K562 cells or immobilized anti-CD16 produced a more robust response than seen in pre-VTX-2337 blood samples. NK cells from Patient 1, which were initially refractory prior to VTX-2337 administration, became responsive to both NKG2D and FcγRIII signaling following VTX-2337 treatment. For Patient 2 where the NK cells from the pre-VTX-2337 blood sample were initially responsive to NKG2D and FcγRIII activating signals; the response was further augmented by VTX-2337 treatment.

## Discussion

VTX-2337 is a potent TLR8 agonist that is currently in Phase 2 clinical development as an immunotherapy for multiple cancer indications, including SCCHN. VTX-2337 has been shown to stimulate monocytes and mDC to produce Th1-polarizing cytokines, activate NK cells, and enhance tumor directed ADCC [3, 20]. An additional—and unexpected—activity of VTX-2337 observed in the current study was the induction of high levels of mature, secreted IL-1β and IL-18 in the absence of additional activation signals. This study therefore presents a cohesive model of NK cell activation by VTX-2337, which includes NLRP3 activation and the release of IL-18 from TLR8 expressing cells. The actions of IL-18 complement other NK cell stimuli, such as NKG2D ligands and FcγRIII activation by therapeutic mAbs, which engage immune cells in ADCC.

The biology of the IL-1 cytokine family is intimately linked to the activation of the TLR family by pathogen-associated molecular patterns such as lipopolysaccharide and single stranded RNA. Agonists of multiple TLRs have been shown to induce the synthesis and subsequent accumulation of pro-IL-1β and pro-IL-18 [21]. When THP-1 cells were treated with VTX-2337, there was a dose-dependent induction of both IL-1β and IL-18 mRNA (Fig 2A and 2B). However, the release of mature IL-1 family cytokines requires the cleavage of pro-cytokines by activated caspase-1, a component of the NLRP3 inflammasome. NLRP3 activation requires a second signal, generally associated with perturbations in normal cell physiology such as ATP release, uric acid crystal-induced damage, reactive oxygen species, or alterations in lysosome integrity [9–10], as opposed to binding to a specific ligand. Interestingly, VTX-2337, in the absence of any other stimulatory signals, activates NLRP3, resulting in the cleavage and release of soluble IL-1β and IL-18. This signal cascade was shown to function in both the THP-1 cell line, and in monocyte populations present in PBMC from healthy donors (Figs 2 and 3).

The release of mature IL-1 family members from VTX-2337-activated THP-1 cells was dependent on NLRP3, as demonstrated by inhibition of caspase-1 with z-VAD, (Figs 2E and 2F, 4A and 4B). In contrast, the NLRP3-independent TNFα response was not impacted by caspase-1 inhibition, as shown in Fig 4D. Previous studies have shown that VTX-2337 stimulates IFNγ production from NK cells and that IL-18 is an important co-regulator of this response [3]. Experiments described in this report confirm the role of IL-18, as anti-IL-18 reduces IFNγ production and CD107a expression (Fig 4E and 4F), while caspase-1 inhibition leads to a marked reduction in IFNγ secretion induced by VTX-2337 (Fig 4C and 4D). Collectively, these results demonstrate dual TLR8 and NLRP3 activation by VTX-2337 in mDC and monocytes populations, and NK cell activation through the downstream release of IL-18.

Mechanisms that underlie NLRP3 inflammasome activation by VTX-2337 are under investigation, but may result from its lipophilic, basic amine structure. These chemical features allow the molecule to concentrate in the low pH environment of the lysosomes. Perturbation of organelle physiology leads to cathepsin B release and activation of NLRP3 [22]. When THP-1 or PBMC cells are pre-treated with the cathepsin B inhibitor, CA-074-Me, VTX-2337 was no longer able to stimulate the release of IL-1β (data not shown). This result is consistent with VTX-2337 providing the signal for the production of pro-IL1β and pro-IL-18 proteins via TLR8, while other features of the molecule activate NLRP3 to mediate the release of mature cytokines.

NLRP3 inflammasome activation by VTX-2337 is not limited to *in vitro* systems or the result of high concentrations that cannot be achieved *in vivo*. The SC administration of VTX-2337 to cynomolgus monkeys resulted in a dose-dependent increase in plasma levels of IL-1β and IL-18, as well as IL-6, a TLR8-induced cytokine that is not dependent on NLRP3 activation (Fig 6). Additionally, the administration of VTX-2337 induced a transient increase in plasma IFNγ levels, consistent with results from in vitro studies showing NK cell activation due in part to the actions of IL-18 and IL-12, as previously reported [3].

While TLR8-inducible mediators, including IL-12 and IL-18, act cooperatively on NK cells, additional signals including tumor-expressed NKG2D ligands [4], and FcγRIII activation via ADCC, intensify the response. This paradigm for NK cell activation, where cytokine priming occurs in conjunction with signaling transduced by activating cell surface molecules, was demonstrated with cells from healthy volunteers (Fig 5C and 5D). It is recognized that cancer patients may have reduced NK cell function, which compromises their capacity to recognize and destroy abnormal tumor cells [23, 24]. In SCCHN, cetuximab is frequently included as “standard of care”, and therapeutic responses achieved with this mAb correlate with FcγRIII activation and enhanced ADCC activity [5]. Cetuximab-activated NK cells have been shown to have additional effector activities, including the production of IFNγ [6], which can also be augmented by VTX-2337 [20]. Thus the capacity of VTX-2337 to enhance NK cell function provides a strong rationale for evaluating this immunotherapy in SCCHN patients who are receiving cetuximab.

In a clinical trial of VTX-2337 in patients with SCCHN, NK cells from peripheral blood were assessed for activation markers. Prior to VTX-2337 dosing, NK cells from one patient were poorly responsive to *ex vivo* activation, while a second patient showed moderate activation (Fig 7). Following VTX-2337 treatment, both patients had robust NK cell responses to either the K562 target cells or to FcγRIII activation by immobilized anti-CD16. These data support the hypothesis that TLR8 activation primes NK cell responses in cancer patients. Additional activating signals, such as ligands for some NK cell receptors, or the engagement of FcγRIII during ADCC, as would occur with the administration of cetuximab to SCCHN cancer patients, complement the effects of TLR8 activation.

The activation of NK cells through the actions of VTX-2337 may facilitate the development of an adaptive, tumor-directed immune response, with the potential for long-term cancer remission. Enhanced NK cell: dendritic cell cross-talk and subsequent priming of antigen-specific CD8 T cells has been demonstrated *in vitro* by Stephenson *et al*. [20]. TLR8 activation also induces IL-12 from mDC and IFNγ from NK cells, which work cooperatively with IL-18 release to drive Th1 cell development [25]. The production of these mediators, together with increased antigen processing and presentation by TLR8-activated accessory cells [3, 26, 27], set the stage for a seamless transition from NK cell-mediated tumor killing to the development of a tumor antigen-specific cellular immune response.

In summary, while previous studies have demonstrated NK cell activation by TLR agonists [28–30], this study elucidates a novel mechanism of action for VTX-2337, involving both the production of pro-IL-1β and pro-IL-18 and activation of NLRP3. The release of IL-18 and other TLR8-induced mediators augment NK cell activation signals in cancer patients. Preliminary results from a recently completed Phase 1 study in SCCHN patients suggest that VTX-2337 “primes” NK cell function. Enhanced NK cell-mediated lysis of tumor cells should complement other TLR8 responses, facilitating the development of a durable, tumor-specific, adaptive immune response.

## Supporting Information

S1 Checklist TREND checklist for Coordinated Activity of Toll-like Receptor 8. (TLR8) and NLRP3 by the TLR8 Agonist, VTX-2337, Ignites Tumoricidal Natural Killer Cell Activity. (PDF)

S1 Fig VTX-2337-induced activation of CD56^dim^ and CD56^bright^ NK cells. (A) The expression of IFN-γ in CD56^dim^ NK cells; (B) The expression of IFN-γ in CD56^bright^ NK cells; (C) The expression of CD107a in CD56^dim^ NK cells; (D) The expression of CD107a in CD56^bright^ NK cells. Each data point represents an individual donor (n = 11 donors). The horizontal bar represents group average. Each donor was analyzed in an independent experiment. **, *p*<0.01, ***, *p*<0.001 by Wilcoxon signed-rank test.
(PDF)

S1 File Data sets from experiments and studies used in presented figures. (XLSX)

S1 Protocol Study Protocol, VTX-2337 Phase 1 Trial in SCCHN_Protocol_A103. (PDF)
