# Supplementary material for: Coordinated Activation of Toll-Like Receptor8 (TLR8) and NLRP3 by the TLR8 Agonist, VTX-2337, Ignites Tumoricidal Natural Killer Cell Activity
Source: PLoS One. 2016 Feb 29;11(2):e0148764. doi: 10.1371/journal.pone.0148764 (PMC4771163; doi:10.1371/journal.pone.0148764)
Supplement: S1 Fig — (A) The expression of IFN-γ in CD56dim NK cells; (B) The expression of IFN-γ in CD56bright NK cells; (C) The expression of CD107a in CD56dim NK cells; (D) The expression of CD107a in CD56bright NK cells. Each data point represents an individual donor (n = 11 donors). The horizontal bar represents group average. Each donor was analyzed in an independent experiment. **, p<0.01, ***, p<0.001 by Wilcoxon signed-rank test. (PDF) [file pone.0148764.s002.pdf]

S1 Fig.

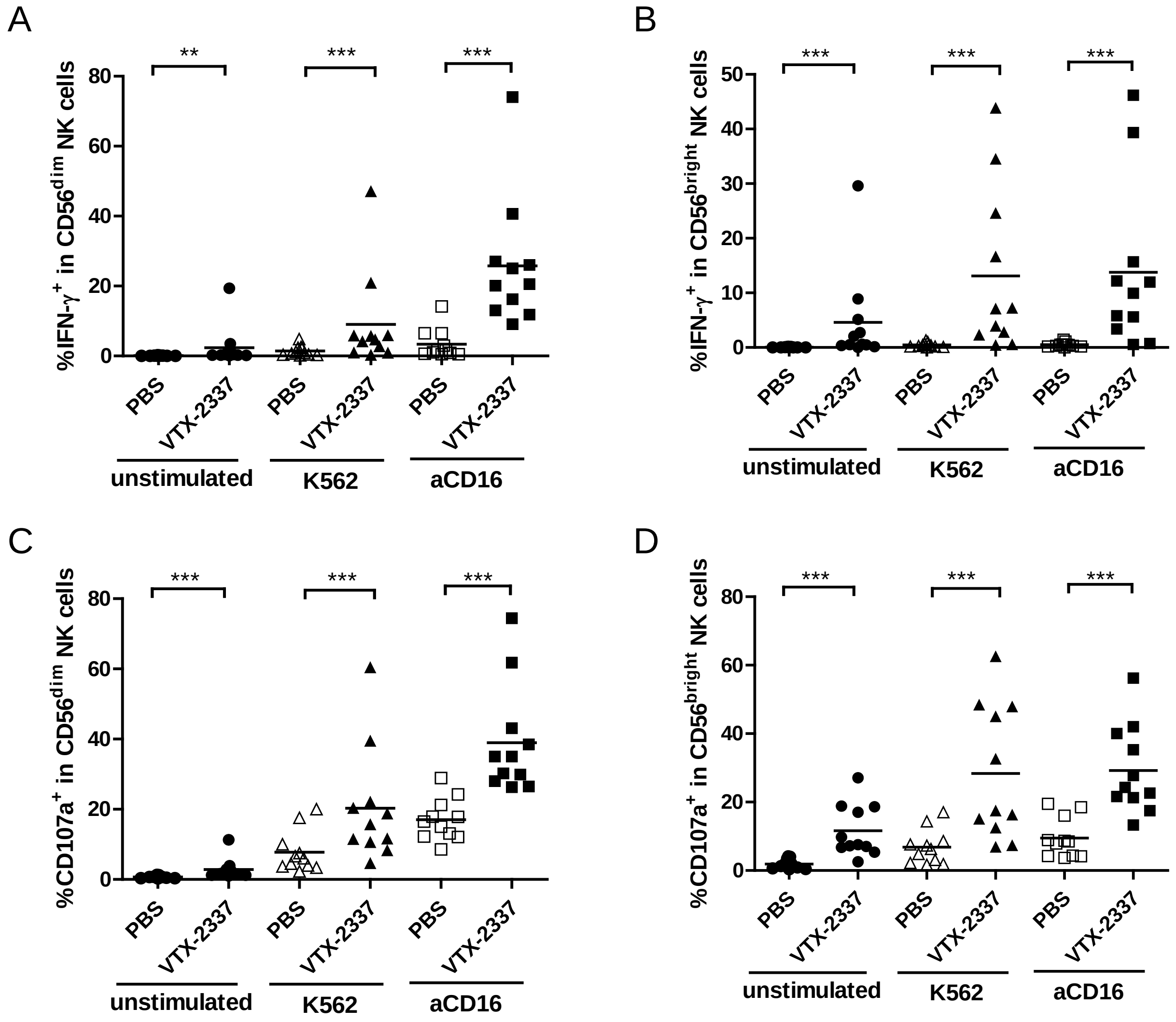

S1 Fig. VTX-2337-induced activation of $CD56^{dim}$ and $CD56^{bright}$ NK cells (A) The expression of IFN-γ in $CD56^{dim}$ NK cells; (B) The expression of IFN-γ in $CD56^{bright}$ NK cells; (C) The expression of CD107a in $CD56^{dim}$ NK cells; (D) The expression of CD107a in $CD56^{bright}$ NK cells. Each data point represents an individual donor (n= 11 donors). The horizontal bar represents group average. Each donor was analyzed in an independent experiment. **, p<0.01, ***, p<0.001 by Wilcoxon signed-rank test.
