# Supplementary material for: Coordinated Activation of Toll-Like Receptor8 (TLR8) and NLRP3 by the TLR8 Agonist, VTX-2337, Ignites Tumoricidal Natural Killer Cell Activity
Source: PLoS One. 2016 Feb 29;11(2):e0148764. doi: 10.1371/journal.pone.0148764 (PMC4771163; doi:10.1371/journal.pone.0148764)
Supplement: S1 Protocol — (PDF) [file pone.0148764.s004.pdf]

IRB Approved
Document Release Date: 05/27/2014

7406

Clinical Study Protocol

Phase I Clinical Trial of VTX-2337, a small molecule Toll-Like Receptor 8 (TLR8) agonist in combination with cetuximab in patients with recurrent or metastatic squamous cell carcinomas of the head and neck (SCCHN)

Sponsor Investigator: Laura QM Chow[1]

Co-Investigators:
Renato Martins[1]

Collaborators:
Nora Disis[2], Robert Hershberg[3]

[1]Clinical Investigators at the University of Washington/FHCRC, [2]Translational Scientist at University of Washington/FHCRC, [3]Director, Executive Vice President and Chief Medical Officer at VentiRx Pharmaceuticals Inc.

Draft Date: 03.11.2014

Printed on 10/21/2015

TABLE OF CONTENTS

1.0 SUMMARY 4

2.0 BACKGROUND 5

3.0 STUDY POPULATION AND SELECTION OF SUBJECTS 13

4.0 TRIAL DESIGN AND TREATMENT PLAN 15

5.0 STUDY CONDUCT AND TRIAL PROCEDURES 24

6.0 DATA ANALYSIS/STATISTICAL METHODS 40

7.0 ASSESSMENTS 42

8.0 REGULATORY, QUALITY AND ADMINISTRATIVE REQUIREMENTS 45

9.0 SUPPLEMENTS 48

LIST OF TABLES

Table 1: Probability of Escalation to the Next Dose for Each True Underlying DLT Rate at a Dose Level 40

Table 2: Probability of Failing to Observe True Underlying DLT Rate at a Dose Level 40

Table 3: Response Evaluation Criteria in Solid Tumors 58

LIST OF FIGURES

Figure 1: Treatment Schema 23

Figure 2: Treatment Algorithm in Case of Cetuximab-related Skin Toxicity 35

1.0 SUMMARY

Title

Immunologic Correlative Studies for a Phase I Clinical Trial of VTX-2337, a small molecule Toll-Like Receptor 8 (TLR8) agonist in combination with cetuximab in patients with recurrent or metastatic squamous cell carcinomas of the head and neck (SCCHN)

Indication

Recurrent or metastatic squamous cell carcinomas of the head and neck appropriate for treatment with cetuximab.

Objectives

Primary Objective: The primary goal of this phase I clinical study is to determine the safety, tolerability and to assess the principal toxicities of VTX-2337 when given in conjunction with cetuximab in order to define the MTD/RP2D.

Secondary Objective: The secondary objective of this phase I clinical trial is to determine the pharmacodynamic response of VTX-2337 in combination with cetuximab.

This protocol will enable the exploration of correlative immune and biologic markers using patient serum and tissue to determine the immunologic response and biological activity of VTX-2337 in combination with cetuximab. Primary assays will involve assessments for VTX-2337 stimulation of serum-induced cytokine responses. Other assays will be descriptive assessments of immune response through peripheral blood and tumor tissue analysis.

Exploratory Objective: An additional tertiary objective will be to conduct a preliminary assessment of anti-tumor activity of cetuximab and VTX-2337 when co-administered to patients with recurrent or metastatic SCCHN.

Study Length

Anticipated first patient in: April 2011

Anticipated last patient out: July 2013

Expected date of Final report: December 2013

Duration of Treatment Period: 24 to 30 months recruitment and treatment, 6 months follow-up.

Number of patients to be enrolled: anticipated 12 to 15 patients, maximum 18 patients.

2.0 BACKGROUND

Head and neck carcinomas (HNCs) are the fifth most common cancer in the world, with increasing incidence.[1, 2] In the United States, over 40,000 to 50000 new cases of HNC are diagnosed yearly.[2] HNCs describe malignancies of the upper aerodigestive tract which include squamous cell carcinomas of the oral cavity, nasopharynx, pharynx and larynx. Although independently, tobacco and alcohol use are the most common risk factors for HNCs, combined use substantially increases the risk of malignancy.[1-4] Occupational risk factors include nickel refining, woodworking and exposure to textile fibers.[1-4] Epstein-Barr virus and human papilloma virus may also play a role in the development of carcinomas of the nasopharynx and squamous cell carcinomas of the oropharynx, respectively.[5, 6]

Despite advances in the diagnosis and treatment of HNCs over the years, the 5 year survival rate over the last few decades remains poor.[3] Although early stage (stage I or II) SCCHN can be cured with surgery and/or radiation therapy, recurrent disease and new primary disease can render patients refractory to the above curative modalities.[7, 8] Moreover the majority of SCCHN cases (up to 75%) are diagnosed in its late stages (stages III or IV).[9, 10] Although some locally advanced carcinomas can be still treated with curative intent with surgery and/or concurrent chemotherapy and radiation therapy, the overall 5 year survival rate is less than 30%.[11] Over 60% of patients relapse within 2 years of initial treatment.[11, 12] Over 10% of patients present with distant metastases and many patients will ultimately develop metastatic disease.[13] The worldwide impact of SCCHN is high, and the morbidity and mortality due to this disease is substantial. The overall prognosis is poor and better therapies are definitely needed for this serious disease.

Metastatic and recurrent SCCHN that is no longer amenable to local surgical/radiation therapy causes substantial morbidity and high mortality with a median survival of 6 to 9 months due to the lack of effective therapeutic options.[11-13] Although first-line platinum-based therapy confers response rates of 15 to 30% with response durations of 3 to 5 months, it involves significant toxicity and many patients are platinum-intolerant or become platinum-refractory.[14] For platinum-intolerant, platinum-refractory or SCCHN patients failing first-line therapy, the response rate to chemotherapeutic agents remains low (< 5%) with a dismal median time to progression (< 3 months).[7, 14] As more than 90% SCCHNs express the epidermal growth factor receptor (EGFR), targeting EGFR and its signaling pathway significantly advanced the treatment of SCCHN.[15] The EGFR-targeted IgG1 monoclonal antibody, cetuximab, became approved for use in recurrent or metastatic platinum-intolerant or platinum-refractory SCCHN patients by the United States Food and Drug Administration (FDA), based on its demonstrated clinical benefit and tolerability in the phase II setting.[16-18] However, in this difficult-to-treat SCCHN population, the observed responses of 13% and a median time to progression of 70 days for cetuximab were certainly not ideal.[16-18] Therefore, combinations that enhance response and survival, without adding increasing toxicity, are still highly needed.

Toll-like receptors (TLR) are important mediators in the immune response. TLR agonists can have an immunostimulatory effect and anti-tumor activity.[19-21] TLR agonists induce a potent innate and adaptive immune response and enhance the antibody dependent cell mediated cytotoxicity (ADCC) of monoclonal antibodies.[19-21] This preclinical potent immune anti-cancer effect is particularly pronounced in SCCHNs.[21, 22] VTX-2337 is a small molecule TLR8 agonist, developed by VentiRx Pharmaceuticals Inc. This novel agent stimulates myeloid dendritic cells and monocytes, and enhances NK cell activity to elicit an immune response to destroy cancer cells.[23, 24] Administered subcutaneously on a weekly basis for three out of four weeks, the phase I single agent dosing of VTX-2337 has demonstrated immunostimulatory activity with minimal toxicity in patients with advanced solid malignancies.[23, 24]

In-vitro studies conducted have demonstrated that VTX-2337 augments natural killer (NK) cell function and in combination with cetuximab enhances antibody-dependent cell-mediated cytotoxicity (ADCC).[24] The combination results in enhanced ADCC both in individuals with high affinity Fc receptors as well as those individuals with a single nucleotide polymorphism (SNP) associated with a suboptimal clinical response to monoclonal antibody treatment.[24] Preclinical studies of TLR agonists demonstrated substantial anti-tumor effects in combination with EGFR agents: TLR agonists independently impaired EGFR signaling and demonstrated potent synergistic anti-tumor activity in combination with EGFR inhibitors such as cetuximab in GEO-CR colon cancer xenografts.[19, 20] In fact, the synergy observed with the addition of TLR agonists was partially due to the enhancement of the ADCC-mediated effects of cetuximab.[19, 20] However, as combined therapy was also effective in anti-EGFR-resistant tumors in an ADCC-independent manner, systemic immune responses stimulated by TLR agonists also appeared to play a role in the anti-tumor effect.[19, 20] As of yet, the full anti-tumor effects of TLR in combination with cetuximab have not yet been fully elucidated; however, the combination of TLR agonists with cetuximab is an exciting high- priority combination for early exploration in SCCHN. As VTX-2337 may enhance therapeutic responses of cetuximab, this combination should be explored for its potential for improved anti-tumor effects in this much needed population. A phase I first-in-human clinical trial of the combination of VTX-2337 and cetuximab will be conducted to explore the combined toxicities, to assess the safety and tolerability, and to establish a maximum tolerated dose (MTD)/recommended phase II dose (RP2D). Importantly, this clinical trial will enable exploration of crucial correlative biologic markers of VTX-2337 immune response in combination with cetuximab. Correlative analysis of patient serum and tissues for immunostimulatory will allow further insight into VTX-2337's mode of action, characterize the immunologic responses elicited by this combination, and explore the biologic immune markers that may predict therapeutic response. Finally, this study will allow exploration of the preliminary anti-tumor activity of this combination in SCCHN.

2.1 Medical Rationale

Head and neck carcinomas are the fifth most common cancer in the world, with increasing incidence due to their strong association with tobacco use and alcohol consumption.[1, 2] Metastatic and recurrent SCCHN that is no longer amenable to local surgical/radiation

therapy, causes substantial morbidity and high mortality with a median survival of 6 to 9 months due to the lack of effective therapeutic options.[3, 6] Although first-line platinum-based therapy confers response rates of 15 to 30% with response durations of 3 to 5 months, it involves significant toxicity and many patients are platinum-intolerant or become platinum-refractory.[7, 14] For platinum-intolerant, platinum-refractory or SCCHN patients failing first-line therapy, the response rate to chemotherapeutic agents remains low (< 5%) with a dismal median time to progression (< 3 months).[7, 14] As more than 90% SCCHNs express the epidermal EGFR, targeting EGFR and its signaling pathway significantly advanced the treatment of SCCHN.[15] The EGFR-targeted IgG1 monoclonal antibody, cetuximab, became approved for use in recurrent or metastatic platinum-intolerant or platinum-refractory SCCHN patients by the United States Food and Drug Administration (FDA), based on its demonstrated clinical benefit and tolerability in the phase II setting.[16-18] However, in this difficult-to-treat SCCHN population, the observed responses of 13% and a median time to progression of 70 days for cetuximab were certainly not ideal.[16-18] Therefore, combinations that enhance response and survival, without adding increasing toxicity, are still highly needed.

VTX-2337 is a small molecule toll-like receptor (TLR) 8 agonist, developed by VentiRx Pharmaceuticals Inc. The TLRs are pattern recognition receptors involved in the innate immunity pathway that trigger the initiation of defense mechanisms and serve as a link between the innate and adaptive immune responses.[21, 22] These new class of agents are being explored for their potent preclinical anti-cancer effects, particular in SCCHN.[21, 22] This novel agent stimulates myeloid dendritic cells and monocytes, and enhances NK cell activity to elicit an immune response to destroy cancer cells.[24] The addition of VTX-2337 may enhance the anti-tumor effects of targeted compounds such as cetuximab. TLRs have demonstrated remarkable synergy with EGFR inhibitors in preclinical colon-cancer models through down regulation of EGFR signaling, induction of apoptosis, antigen-dependent cellular cytotoxic - mediated effects and separate not yet fully elucidated immune-related anti-cancer effects.[19, 20] The synergy of TLR agonists with the EGFR monoclonal antibody, cetuximab, is remarkable preclinically.[19, 20] As previously noted, in vitro studies have demonstrated that VTX-2337 augments natural killer (NK) cell function, and in combination with cetuximab enhances antibody dependent cell mediated cytotoxicity (ADCC).[24] As such, VTX-2337, may enhance the therapeutic responses of cetuximab, and this combination should be explored for its potential to improve anti-tumor efficacy in this population. Administered subcutaneously on a weekly basis for three out of four weeks, the phase I single agent dosing of VTX-2337 has demonstrated immunostimulatory activity with minimal toxicity in patients with advanced solid malignancies.[24]

A phase I first-in-human clinical trial of the combination of VTX-2337 and cetuximab will be conducted to explore the toxicities of the combined regimen, to assess the safety and tolerability, and to establish a maximum tolerated dose (MTD)/recommended phase II dose (RP2D). Importantly, this clinical trial will enable exploration of crucial correlative biologic markers of VTX-2337 immune response in combination with cetuximab. Correlative

analysis of patient serum and tissues for immunostimulatory will allow further insight into VTX-2337's mode of action, characterize the immunologic responses elicited by this combination, and explore the biologic immune markers that may predict therapeutic response. Finally, preliminary anti-tumor activity of this combination in SCCHN will be explored.

2.2 Investigational Drug VTX-2337

Background

The immune system has a significant role to play in the emergence of malignancy. When immune surveillance fails to detect and destroy abnormal cells, malignancy develops. In particular, when the immune system fails to mount a T cell mediated cellular response to tumor associated antigens and tolerance develops, tumors persist and grow. By augmenting and stimulating the immune response against cancer, there may be clinical anti-cancer activity.

Immunity involves the innate and adaptive immune responses. Adaptive responses produce cellular responses with antigen-specific B and T lymphocytes that take days to weeks to develop; whereas, innate immune responses involve acute, inflammatory responses to microbial or endogenous antigens and are rapid. The innate immune response utilizes a variety of target cells and receptors: pattern recognition receptors (PRR) are employed that recognize invariant structures within microbial agents.[21, 22] Such recognition by PRRs, including TLRs, triggers the rapid production of chemokines, cytokines, and other inflammatory mediators that leads to the generation of cellular immune responses.[21, 22] Augmenting the innate immune response in patients with cancer may reduce immune tolerance by targeting the tumor microenvironment (for example, by activating tumor macrophages and dendritic cells) and by enhancing antigen-specific T cell responses.[21, 22]

Activation of the innate immune response in humans is regulated in part by TLRs. TLRs are a family of ten evolutionarily conserved receptors expressed either on the cell surface or in intracellular endocytic vesicles or organelles, which recognize a diverse range of microbial ligands. TLR agonists induce NK cell activity, enhance ADCC, induce cytokines and chemokines, and modulate T cell responses. In addition, TLR agonists may decrease regulatory T cells activity.[21, 22] Overall, the immunomodulatory effects of TLR agonists render them attractive as anti-cancer agents, particularly in SCCHN, where potent anti-cancer effects have been observed preclinically.[21, 22]

VTX-2337, a novel small molecule TLR8 agonist, developed by VentiRx Pharmaceuticals Inc, has been shown preclinically to stimulate myeloid dendritic cells and monocytes, and enhances NK cell activity and ADCC to elicit an immune response to destroy cancer cells.[23, 24] In a phase I first-in human clinical trial involving VTX-2337 alone, doses were escalated from 0.1 mg/m^2 to 3.9 mg/m^2 on an administration schedule of an once-weekly subcutaneous injection for 3 out of 4 weeks.[24] Overall, this treatment was well-tolerated

with the primary drug-related side effects of chills, fatigue, influenza-like illness, injection site reactions, and pyrexia. Only one incidence of Grade 3 dose-limiting hypotension was observed in one patient at the top dose level, and this was transient and reversible.[24] The pharmacokinetic profile has well documented, dose-dependent increases in Cmax and AUC, and pharmacodynamic measurements using a panel of immune biomarkers demonstrated dose-dependent increases in a variety of inflammatory mediators.[24]

2.2.1 API and Formulation

VTX-378 is a novel small molecule Toll-like receptor (TLR) 8 agonist. Initial chemical characterization identifies VTX-378 as a small organic molecule with a molecular weight of 458.6.[24] This active pharmaceutical ingredient (API) is formulated in Captisol®, a cyclodextrin-based solubilizing agent, to produce the investigational drug VTX-2337. Lyophilized VTX-2337 is supplied in sterile single-use vials, and is reconstituted with Sterile Water for Injection, prior to subcutaneous administration at a dose volume of ≤ 1.0 mL. The Investigator Brochure gives additional information.[24]

2.2.2 Summary of Non-Clinical Findings of Relevance to the Study

More extensive information regarding the pre-clinical pharmacology and toxicology of VTX-2337 may be found in the Investigator's Brochure.[24]

VTX-2337 is a selective agonist for TLR8, which is expressed in the endosomal compartment of monocytes and myeloid dendritic cells (mDC). The target for VTX-2337, therefore, is monocyte and mDC populations in the peripheral blood, lymphoid organs, and within the tumor microenvironment. Murine cells do not respond to synthetic or natural ligands to TLR8, precluding the use of traditional xenograft models to explore the potential in vivo efficacy of TLR8 agonists in cancer. Accordingly, preclinical activity was focused on defining the biological activity of VTX-2337 in cynomolgus monkeys and correlating these activities with human cells in vitro.[24]

Pharmacokinetics and Pharmacology

In cultures of human peripheral blood mononuclear cells (PBMC), VTX-378 (the API) induces a variety of pro-inflammatory mediators in a time- and dose-dependent manner. These mediators include multiple chemokines (e.g., MCP-1, MIP-1β) and cytokines (e.g., IL-6, IL-1, TNFα, IL-12). The selectivity of this compound has been assessed in PBMC and in cells engineered to express only a single TLR. In both PBMC and cell transfectants, VTX-378 is highly selective for TLR8 with an EC_{50} of approximately 70 nM. VTX-378 shows minimal activity for TLR7 (with an EC_{50} of approximately 3 μM) but does not stimulate other TLRs.[24]

The pharmacology of VTX-2337 has been extensively evaluated in cynomolgus monkeys. The compound shows linear pharmacokinetics over a wide dose range, with a $t_{1/2}$ of approximately 2-4 hours. The administration of VTX-2337 in monkeys elicits the production

of multiple inflammatory mediators which can be readily measured in plasma, and which closely mirror the mediators seen following in vitro stimulation of PBMC with VTX-378. A clear pharmacokinetic-pharmacodynamic relationship is observed for many of the cytokines, chemokines, and other inflammatory markers produced. Administration of VTX-2337 also results in transient changes in blood cell populations consistent with cellular activation of neutrophils, NK cells, and T cells, and may result in the transient extravasation of these latter cell populations from the blood compartment.[24]

Toxicology

Standard acute and repeated dose toxicity studies of VTX-2337 were conducted in mice and cynomolgus monkeys. Findings from the 28-day repeat dose studies in cynomolgus monkeys are most relevant to this clinical protocol, and are therefore summarized here.

Administration of VTX-2337 to cynomolgus monkeys has resulted in significant toxicity, including generalized constitutional symptoms and inflammation of the eyes (uveitis and retinitis), most notably at higher doses. The systemic toxicities seen at higher doses included pyrexia and clinical observations such as hypoactivity and anorexia, which appear consistent with the flu-like response frequently seen with immunomodulatory agents. The development of ocular inflammation appears to be mechanism based, and published reports have implicated several chemokines and cytokines induced by VTX-2337 (e.g., IL-6, IL-8, and MCP-1) in the pathogenesis of uveitis. While the incidence and severity of uveitis was found to generally increase with the dose level of VTX-2337 and the number of weekly doses administered, there was evidence of reversibility when dosing was terminated. In addition, periodic ophthalmologic examinations were able to identify inflammatory changes relatively early, before they progressed to more serious lesions from continued dosing. Of note, the first clinical study of VTX-2337—in patients with advanced solid tumors—required that a comprehensive, dilated ophthalmologic examination be performed after each dose of study drug. No drug-related ophthalmologic toxicities were observed in the 33 subjects treated with VTX-2337 at dose levels of 0.1 up to 3.9 mg/m^2.[24]

In summary, the standard battery of genetic toxicology, safety pharmacology, acute and repeated dose toxicity studies were conducted in compliance with FDA Good Laboratory Practice guidelines and provided the basis for the phase I first-in human study of VTX-2337 alone which has been completed.

2.2.3 Clinical Findings of Relevance to the Study

Study VRXP-A101 was the first clinical study of VTX-2337. This Phase I clinical study identified the MTD of single-agent VTX-2337.[24] VTX-2337 was administered as a ≤ 0.3 mL subcutaneous injection on Day 1, Day 8, and Day 15 of a 28-day cycle. Eligible subjects received two cycles of this regimen (i.e., 6 doses of VTX-2337). In some instances, subjects whose tumors demonstrate a complete response, partial response, or stabilization of disease on Week 8 imaging studies were eligible to receive additional

cycles of VTX-2337. The first cohort of subjects received 0.1 mg/m^2 of VTX-2337 per dose according to the regimen described above. The dose level was increased for successive cohorts with a dose-escalation plan in a standard 3 + 3 design if no dose-limiting toxicities (DLTs) were observed: 0.1mg/m^2, 0.2mg/m^2, 0.4mg/m^2, 0.8mg/m^2, 1.3mg/m^2, 2.0mg/m^2, 2.8 mg/m^2 and 3.9 mg/m^2. At 3.9 mg/m^2, one of six evaluable subjects experienced a DLT of Grade 3 hypotension. This patient recovered completely with no sequelae. Accordingly, the maximum-tolerated dose (MTD) was the highest dose level evaluated (3.9 mg/m^2) in this study. No other DLTs were noted during the study. Although 3.9 mg/m^2 was determined to be the MTD, the recommended phase II dose (RP2D) of VTX-2337 in combination with cetuximab is anticipated to be between 2.0 mg/m^2 to 3.5 mg/m^2, based on the toxicity profile, pharmacokinetics and pharmacodynamic indicators of immune response.

2.2.4 Potential Risks

VTX-2337 is a potent immunomodulatory compound that stimulates TLR8. Various TLR agonists have been administered to humans in the oncology and infectious disease settings. In general, these agents are reasonably well-tolerated. The most frequently observed drug-related toxicities can be characterized by localized (e.g., injection site) or generalized (e.g., chills, fatigue, influenza-like illness, pyrexia) findings. In addition, infrequent but medically important events of cytokine release syndrome and hypotension were observed in the Phase 1 study.[24]

Potential long-term clinical effects of VTX-2337 cannot be predicted based on the current toxicology studies which extend to 28 days. However, eight subjects entered an extended dosing protocol, with several subjects receiving more than 6 cycles (18 individual weekly doses) without problems.

Overall, at the dose levels anticipated to be administered in this clinical trial, VTX-2337 treatment-related toxicities are expected to be those events commonly associated with an activated immune response. This includes symptoms of chills, fever, flu-like syndrome, headache, and local injection site reaction.

2.3 Cetuximab

Background

The EGFR is highly expressed (>90%) in SCCHN.[25] Preclinically the inhibition of the EGFR signaling pathway with a monoclonal antibody has demonstrated substantial tumor regressions.[26] Cetuximab (Erbitux®, C-225) is a G subclass (IgG1) chimeric antibody, genetically engineered from the variable region of the heavy and light chains of the murine monoclonal antibody M225 and the constant regions of the human kappa light chain of the gamma1 heavy chain.[27] Cetuximab has higher affinity for the EGFR than the epidermal growth factor (EGF) and other binding ligands. It competitively binds to EGFR to inhibit

ligand-induced activation of EGFR cell signaling pathways, and results in internalization of the EGFR.[27]

2.3.1 Clinical Findings of Relevance to the Study

A number of clinical studies have demonstrated the utility and safety of cetuximab in the treatment of SCCHN. In an open label Phase 2 study involving 103 patients with platinum-refractory recurrent or metastatic SCCHN, cetuximab alone (administered IV with a 400mg/m^2 initial loading dose followed by 250mg/m^2 weekly doses), demonstrated an objective response rate (ORR) of 13%, a disease control rate of 46% and a median time to progression (TTP) of 70 days.[16, 17] Treatment was well tolerated with the exception of mild rash and infusion-related reactions.[17]

In an analysis of three phase II studies involving cetuximab as monotherapy or plus platinum based regimens, the overall response rates ranged from 10 to 13%, with disease control rates of 46 to 56%, with a median time to progression of 2.2 to 2.8 months and a median overall survival of 5.2 to 6.1 months.[16, 18, 28-31] No patient who responded to cetuximab, responded to additional platinum. Cetuximab was found to have a clinical benefit and overall survival benefit in this refractory population of SCCHN.[16, 28-30] Its clinical activity confirmed in additional phase II studies of cetuximab in SCCHN, led to Phase III trials of cetuximab in combination with chemotherapy and radiation therapy.[18] In 117 patients with recurrent or metastatic SCCHN, randomized to cetuximab plus cisplatin 100mg/m^2 IV q 28 days) compared to cisplatin plus placebo, demonstrated a superior ORR (26% vs. 10%; p=0.03), an improved median progression-free survival (PFS) of 4.2 months vs. 2.7 months (with hazard ratio of 0.78; p=0.09), and an improvement in median OS of 9.2 months vs. 8.0 months (p=0.21) favoring the combination compared to cisplatin monotherapy.[32-34] This study did not achieve the primary endpoint of statistically significantly improved PFS, but the increase in ORR and positive trends to improved PFS and OS suggested a clinical benefit.[32-34]

Another randomized phase III study compared cetuximab plus platinum and 5-fluorouracil (5-FU) to platinum and 5-FU in 442 patients with recurrent or metastatic SCCHN, and demonstrated that the addition of cetuximab to platinum and 5-FU improved the median OS (10.1 months vs. 7.4 months with hazard ratio of 0.80; p=0.04) and median PFS (5.6 months vs. 3.3 months with hazard ratio of 0.54; p<.001).[28, 35] With the exception of skin reactions, the incidence of Grade 3 or greater toxic effects was similar between the 2 groups.[28, 35] Lastly, 424 patients with locoregionally advanced (stages III-IV) SCCHN were randomized to cetuximab plus radiotherapy to radiotherapy alone in a phase III study, which demonstrated an improved median duration of locoregional control (24.4 months vs. 14.9 months with hazard ratio of 0.68; p=0.005), a median PFS (17.1 months vs. 12.4 months with hazard ratio of 0.70; p=0.006), and a median OS (49.0 months vs. 29.3 months with hazard ratio of 0.74; p=0.03) favoring the addition of cetuximab to radiation therapy.[36, 37] With the exception of acneiform rash and infusion reactions, the incidence of Grade 3 or greater toxic effects was similar between the 2 groups.[36, 37] Clearly, cetuximab has been found to be an active and well-tolerated agent for the treatment of SCCHN.

Cetuximab is currently approved by the United States FDA for use in treatment of patients with locally or regionally advanced SCCHN in combination with radiation therapy and recurrent or metastatic SCCHN progressing after platinum-based therapy.[16]

3.0 STUDY POPULATION AND SELECTION OF SUBJECTS

This study will be conducted in adult subjects with advanced recurrent SCCHN that is no longer amenable to treatment by surgery or radiation therapy, or SCCHN patients with distant metastatic disease. Subjects may have failed or become refractory to prior therapies and would be appropriate for cetuximab therapy. Eligible subjects come from a population of patients who are likely to die from their disease, have an average life expectancy of greater than 3 months, have frequently exhausted or refused the armamentarium of available therapies, and for whom few treatment options exist. Specific eligibility requirements will be outlined below.

3.1 Selection of Subjects: Inclusion Criteria

- Patients with a histological or cytopathological confirmed diagnosis of squamous cell carcinoma of the head and neck region that is
  - locally advanced/recurrent and no longer amenable to local surgical or radiation therapy

    and/or
  - has evidence of metastatic disease.
- Patients may have been previously treated with systemic therapy but are otherwise deemed currently platinum-refractory, or would be deemed inappropriate or intolerant to platinum-based chemotherapy.
- Patients must have completed definitive chemotherapy and/or radiation therapy ≥ 3 months prior to study entry.
- Prior therapy with agents targeting/blocking the epidermal growth factor receptor (e.g. cetuximab and erlotinib) is allowable.
- Age ≥ 18 years old.
- Performance Status: ECOG 0 - 2.
- Expected life expectancy of at least 12 weeks, as assessed by the Investigator.
- Ability and willingness to comply with the study's visit and assessment schedule and to provide voluntary written informed consent.
- Acceptable bone marrow, renal, and hepatic function based upon screening lab tests as demonstrated by the following:
  - Absolute neutrophil count (ANC) ≥ 1,500 cells/μL
  - Platelet count ≥ 75,000 cells/μL
  - Hemoglobin ≥ 8.0 g/dL

- Creatinine ≤ 2.0 mg/dL
- Total bilirubin ≤ 2.0 x ULN
- SGOT(AST), SGPT (ALT) ≤ 2.5 x ULN
  (for patients with liver metastases, AST, ALT < 5x ULN is acceptable)

- Willingness to use a medically acceptable method of contraception throughout the study period and for 4 weeks after the final administration of VTX-2337 (all subjects).
- For female subjects with reproductive potential: a negative serum pregnancy test.

3.2 Selection of Subjects: Exclusion Criteria

- Investigational therapy within 4 weeks of study entry.
- Chemotherapy therapy or palliative radiation therapy within the previous 2 weeks prior to dosing with cetuximab or VTX-2337. Patients should have recovered from major toxicities of prior therapy (If deemed reversible, toxicities should return to baseline or ≤ grade 2 in severity).
- Major surgery within the past 4 weeks prior to dosing with cetuximab or VTX-2337.
- Concurrent symptomatic central nervous system (CNS) involvement, brain or leptomeningeal metastases. Treated CNS involvement which has been stable >28 days off systemic steroids may be included.
- Major active psychiatric disorders which would limit compliance.
- Treatment with oral or parenteral corticosteroids within 2 weeks prior to dosing with VTX-2337 or a requirement for systemic immunosuppressive therapy for any reason.
- Active autoimmune disease.
- Clinically significant cardiac disease (e.g., congestive heart failure, unstable or uncontrolled angina, myocardial infarction) within 6 months of dosing with VTX-2337.
- Clinically significant ophthalmologic disease, defined as:
  - Current retinal vascular disorder, including active untreated diabetic retinopathy

    and/or
  - Previous or current uveitis
- Infection requiring parenteral antibiotic therapy or causing fever (temp > 100.5°F or 38.1°C) within 1 week prior to dosing with VTX-2337.
- Pregnant or breast-feeding females.

- Uncontrolled inter-current illness, pre-planned surgery or procedure requiring hospitalization during the study period, or any other condition or circumstance that could interfere with adherence to the study's procedures or requirements, or otherwise compromise the study's objectives.
- Second primary malignancy that is clinically detectable (not including in situ carcinoma of the cervix, non-melanoma skin cancer or low-grade (Gleason score ≤ 6) localized prostate cancer) and demonstrating active progression at the time of consideration for study enrollment.
- Known prior severe allergic/hypersensitivity to cetuximab or any of the components of the study treatment.
- Known prior severe (≥ Grade 3) rash and / or diarrhea toxicities to cetuximab.

4.0 TRIAL DESIGN AND TREATMENT PLAN

4.1 Number of Subjects

Three cohorts of n=3 to 6 subjects will be enrolled. Enrollment of three cohorts is planned. At the MTD or highest dose level in this study, the cohort will be expanded as applicable to ensure a total of 6 to 9 subjects are treated at the RP2D.

***Number of subjects*: ** Maximum of 13 subjects

***Estimated Annual Accrual*:** 12

***Number of Centers*:** 1

4.2 Trial Design

This is a phase I open-label, dose-finding, safety study of VTX-2337 in combination with cetuximab in subjects with advanced recurrent or metastatic SCCHN for which curative therapy is not available. This will be a single center study, un-controlled, non-comparative and non-randomized trial with no stratification.

4.3 Endpoints

Primary Objectives

To determine the safety, tolerability and to assess the dose-limiting toxicities of VTX-2337 when given in conjunction with cetuximab in order to define the maximum tolerated dose (MTD)/recommended phase II dose (RP2D).

Secondary Objectives

To determine the pharmacodynamic immune response to VTX-2337 in combination with cetuximab.

Correlative assessments of immunologic response and activity will be performed, including:

- Quantitative evaluation of baseline immune status via in-vitro assessment of cytokine and chemokine response to immunostimulatory agents
- Quantitative assessment of plasma cytokines, chemokines, and other inflammatory markers via protein array.
- Quantitative assessment of NK cells via flow cytometry.
- Quantitative assessment of antigen-specific responses in cytokine-producing cells to common prognostic SCCHN antigens via INFγ-ELISpot.

Additionally, to assess whether subjects with functional genetic variations in the TLR8 and FCγR IIIA genes have altered biological and/or clinical responses to VTX-2337, genetic characterization of subjects will be performed via standard genotyping assays.

Exploratory Objective

An additional objective will be to assess preliminary evidence of anti-tumor activity for the combination of VTX-2337 and cetuximab, as measured by RECIST v1.1 criteria.

4.4 Dose Rationale

Cetuximab

The initial dose of cetuximab 400 mg/m^2 IV and the subsequent weekly dose of cetuximab 250 mg/m^2 IV are based on the current labeled indication for the treatment of patients with SCCHN in combination with radiation therapy for locally advanced disease, and for recurrent or metastatic disease progressing after platinum-based chemotherapy.[17] This regimen is designed to achieve acceptable serum concentrations saturating >90% of EGFR receptors.[27] Doses of cetuximab will be fixed.

At the investigator's discretion, obese subjects or subjects with high BSAs may be capped at 2.2 mg/m^2.

VTX-2337

From the results of the phase I study of VTX-2337 alone, the MTD was determined to be 3.9mg/m^2. The RP2D is felt to be within the realm of 2.5 mg/m^2 to 3.5 mg/m^2, and the starting dose of VTX-2337 in combination with cetuximab will be at 2.5 mg/m^2. This starting dose will allow assessment of VTX-2337 at active doses without exposing subjects to undue risk of unanticipated toxicity in conjunction with cetuximab. If substantial toxicity occurs at the initial dose level of 2.5 mg/m^2 with DLTs observed in ≥ 2 subjects, the maximal tolerable dose will have been exceeded and subsequent subjects will enroll onto dose level -1 starting at 2.2 mg/m^2.

VTX-2337 will be administered as a ≤ 0.3 mL subcutaneous injection once weekly for three out of four weeks. The first cohort of subjects will receive VTX-2337 at the starting dose level of 2.5 mg/m^2. The dose level will be increased for successive cohorts as delineated in §4.7.

Although data is limited in obese subjects and subjects with a high body surface area (BSA), the body surface area may be capped for VTX-2337 at 2.2 m^2 at the discretion of the Investigator.

4.5 Treatment Regimen

The purpose of this study is to evaluate multiple dose levels of the investigational product VTX-2337 when administered with cetuximab, an approved therapy for the treatment of SCCHN.

The standard dosing of cetuximab involves a loading dose of 400 mg/m^2 followed by 250 mg/m^2 weekly dosing to achieve acceptable serum concentrations.[27] Obese subjects or subjects with high BSAs may be capped at 2.2 mg/m^2 at the treating investigator's discretion. Phase I studies in advanced solid tumors have established the VTX-2337 MTD as 3.9 mg/m^2, and the proposed recommended phase II dose is anticipated to be between 2.0-3.5 mg/m^2 SC for three out of four weeks.[24]

Eligible subjects will be registered in the study and receive either 1 month of cetuximab or a single dose of cetuximab (a loading dose of 400 mg/m^2 will be administered if greater than 3 weeks (21 days) have elapsed since prior cetuximab therapy, otherwise if within 3 weeks of prior cetuximab therapy a dose of 250 mg/m^2 will be given) according to the regimens described above After the cetuximab lead-in period, subjects must meet the criteria as outlined in §5.2 before initiating combined study treatment of cetuximab plus VTX-2337 starting on Day 1 of Week 1.

VTX-2337 will be administered as a ≤ 0.3 mL subcutaneous injection on Day 1, Day 8, and Day 15 of a 28-day cycle. The dose of VTX-2337 will be escalated in a 3+3 phase I design according to §5.7, starting at Dose Level 1.

During combination treatment, cetuximab will be administered first. At the completion of cetuximab dosing, VTX-2337 will be administered subcutaneously and subjects will be observed for at least 15 minutes for acute toxicities before allowing the subjects to leave the infusion unit. After the 4th cycle of combination therapy, if no acute toxicities related to treatment are observed this period is optional.

Although dosing will be strictly administered on the days noted in Cycle 1 for pharmacodynamic studies, subsequent cycles of therapy will allow flexibility (+/- 1 day) as per the Investigator's discretion in the weekly dosing of cetuximab and VTX-2337 for patient convenience/holidays without being considered a protocol deviation. However, every effort should be made to continue to administer cetuximab on a weekly schedule and

continue the administration of VTX-2337 weekly three out of four weeks in subsequent cycles of therapy.

4.6 Administration of Cetuximab

How Supplied and Storage

Cetuximab is supplied in single-use vials containing a 5 mg/mL solution ready to use for IV infusion. No dilution is required.

Cetuximab vials should be stored under refrigeration at 2°C to 8°C (36°F to 46°F) and never frozen. Cetuximab does not contain any antimicrobial preservative or bacteriostatic agent. From a microbiological point of view, the product shall be used immediately after opening. If not used immediately, in-use storage times and conditions prior to use are the responsibility of the user and would normally not be longer than 24 hours at 2°C to 8°C, unless opening has taken place in controlled and validated aseptic conditions.

Dose Preparation

Prior to infusion, the appropriate volume of cetuximab is to be drawn from the vial with a sterile syringe. Cetuximab is to be transferred from the syringe into a sterile evacuated container. Cetuximab will then be filtered through a 0.22-μm protein-sparing or low-protein binding in-line filter. At the end of the infusion, 0.9% normal saline is to be used to flush the line. Different drug product lots must not be mixed in a single infusion.

Dosage and Dose Regimen

Subjects are to receive weekly IV infusions of cetuximab via infusion pump or gravity drip. The 400 mg/m^2 initial dose of cetuximab is administered over 120 minutes. This initial dose is to be followed by weekly infusions of cetuximab 250 mg/m^2 IV over 60 minutes.

The dose and volume of the study medication to be infused are dependent upon the patient's body surface area (BSA). At the treating investigator's discretion, obese subjects or subjects with high BSAs may be capped at 2.2 mg/m^2. The infusion rate should not exceed 10 mg/minute (2.5 mL/minute).

Subjects should be observed closely during the infusion of cetuximab and for 1 hour after an initial 400 mg/m^2 infusion. Availability of resuscitation equipment must be ensured. Prior to an initial 400 mg/m^2 infusion, subjects must receive premedication with an antihistamine (H1 antagonist), such as diphenhydramine hydrochloride (HCl) 50 mg IV. Premedication is recommended for the initial 250 mg/m^2 infusion and prior to subsequent doses; however, the dose of diphenhydramine HCl (or other similar agent) may be reduced or eliminated at the Investigator's discretion. Additional premedication regimens may be used based on the local standard of care. Infusion times may be increased for subjects who experience Grade 1 and 2 infusion reactions; however, the duration of the infusion cannot exceed 4 hours.

Refer also to §5.10.1 for additional instruction regarding management of cetuximab-related toxicity.

4.7 Administration of VTX-2337

How Supplied, Packaging, Labeling and Storage

VTX-2337 is provided as an off-white, lyophilized cake in a 3 mL clear glass, stoppered, single-use vial with a secured rubber cap. Each vial contains 48 mg of the investigational drug, which is comprised of the following:

Active Ingredient:	Quantity (mg/vial):
VTX-378	48
Inactive Ingredients:	
Captisol	180
Citric Acid, Monohydrate	2.3

Each vial is identified with the following label:

VTX-2337 48 mg<br>LN: VEN80005<br>Subject #________________<br>Date: __________________	VTX-2337 powder for subcutaneous injection.<br>Must reconstitute with SWFI and dilute with 0.9% sodium chloride.<br>See clinical protocol.<br>Store 2–8ºC<br>Caution: New drug—limited by federal law to investigational use.<br>VentiRx Pharmaceuticals, Inc

Approximate label size: 4” wide x 0.75” high

The Sponsor-Investigator will be provided a bulk supply of VTX-2337 vials for this open-label study. Unopened vials of VTX-2337 should be stored in a refrigerator at 2ºC to 8ºC (36ºF to 46ºF) and protected from light.

Reconstitution and Dilution

Before administration, VTX-2337 must be reconstituted and then further diluted. Investigational drug solutions should be prepared and transferred using aseptic technique in a biological safety cabinet. Vials of VTX-2337 are intended for single use only.

Each vial containing 48 mg VTX-2337 should be reconstituted with 1.0 mL Sterile Water for Injection, USP, to yield a stock solution with a concentration of 40 mg/mL. To reconstitute a vial of VTX-2337, add the sterile water while directing the stream at the lyophilized cake. Swirl the vial to disperse the water throughout the cake, then invert gently

and repeatedly for approximately 2 minutes until the contents are fully dissolved. The reconstituted stock solution should be clear, and will range in color from colorless to slightly yellow. The solution should be inspected carefully to confirm that no particulates are present. Reconstituted stock solutions are stable for 4 hours at room temperature and for 12 hours when refrigerated at 2°C to 8°C.

Stock solutions of VTX-2337 should be diluted with 0.9% Sodium Chloride Injection, USP (normal saline; must be free of phenol). To prepare the diluted solution for dosing, withdraw the appropriate volume of reconstituted stock solution and transfer to a sterile glass vial. Dilute with the appropriate amount of normal saline to achieve the desired concentration. Serial dilutions may be required to attain the desired concentration. Diluted solutions of VTX-2337 are stable for 4 hours at room temperature and for 12 hours when refrigerated at 2°C to 8°C.

The formulation of VTX-2337 contains no preservatives and is intended for single use only. Vials of stock solution and diluted solution for dosing should be made for each subject and for each dose in the study. Dilutions of VTX-2337 should be drawn from the dilution vial into the syringe just prior to dosing.

Dosage and Dose Regimen

VTX-2337 is administered as a ≤ 0.3 mL subcutaneous injection. Administration will proceed after the Lead-In period (Week -4 to -1 for cetuximab naïve subjects and Week -1 for subjects previously treated with cetuximab). VTX-2337 doses are given weekly for the first 3 weeks (Day 1, Day 8, and Day 15) of a 4-week (28-day) cycle starting Week 1 (Cycle 1, Day 1). Subsequent cycles of therapy should try to continue as weekly administrations of VTX-2337 and cetuximab but will allow flexibility of +/- 1 day for patient convenience and holidays without constituting a protocol deviation. The dose schedule in select instances may be modified at the Investigators discretion to enable subject compliance.

Within 30 minutes prior to each dose of VTX-2337, subjects should be administered 650 mg acetaminophen by mouth to help mitigate potential adverse events commonly associated with the administration of immunomodulatory therapies, such as fever and myalgia. VTX-2337 SC injections will follow the completion of cetuximab infusions and subjects will be observed for 15 minutes post VTX-2337 SC administration for any acute toxicity before being allowed to leave the infusion unit. After the 4th cycle of combination therapy, if no acute toxicities related to treatment are observed this period is optional.

Standard medications to treat possible hypersensitivity reactions should be readily available at the time of treatment, including epinephrine, H1 antihistamines (e.g., diphenhydramine), H2 antihistamines (e.g., ranitidine), and normal saline for volume expansion. Due to their immunosuppressive effect, administration of systemic steroids (e.g., dexamethasone) should be avoided in this setting if other means of treatment are

available and appropriate. Refer also to §5.10.2 for additional instruction regarding management of VTX-2337-related toxicity.

Doses of VTX-2337 should be administered in a volume of ≤ 0.3 mL. The dose of VTX-2337 should be administered with a 1 mL syringe with a ⅝-inch, 23 to 27 gauge needles. Alternatively, a 0.3 mL insulin syringe with a ½-inch, 28-gauge needle can also be used. To administer the injection, 1-2 inches of fatty tissue should be pinched up to avoid injection into the muscle layer. The needle can be inserted at either 45 or 90 degrees; a 45-degree angle is recommended when less than 2 inches of tissue can be pinched. Appropriate anatomic areas for subcutaneous injection include the fatty tissue over the triceps, abdomen, or thighs. Dilutions of VTX-2337 should be drawn from the dilution vial into the syringe just prior to dosing.

Dose levels for VTX-2337 with fixed cetuximab starting in Week 1 (Cycle 1)

Each subject will be assigned to a dose level at the time of registration. The starting dose of VTX-2337 for the first cohort is 2.5 mg/m^2, and escalations in drug dose for successive cohorts will proceed as noted below.

Dose Level -1	• Cetuximab 250 mg/m^2 IV weekly dosing<br>• VTX-2337 2.0 mg/m^2 SC weekly for 3 out of 4 weeks starting in Week 1 (Cycle 1)
Dose Level 1 (starting dose)	• Cetuximab 250 mg/m^2 IV weekly dosing<br>• VTX-2337 2.5 mg/m^2 SC weekly for 3 out of 4 weeks starting in Week 1 (Cycle 1)
Dose Level 2	• Cetuximab 250 mg/m^2 IV weekly dosing<br>• VTX-2337 3.0 mg/m^2 SC weekly for 3 out of 4 weeks starting in Week 1 (Cycle 1)
Dose Level 3	• Cetuximab 250 mg/m^2 IV weekly dosing<br>• VTX-2337 3.5 mg/m^2 SC weekly for 3 out of 4 weeks starting in Week 1 (Cycle 1)

4.8 Treatment Schedule

Study treatment will proceed as per the schema outlined in Figure 1. A lead-in period of either 4 weeks (cetuximab naïve subjects) or 1 week (subjects previously treated with cetuximab) with cetuximab alone will be conducted to assess severe toxicities (see §4.8.1). If severe (≥ Grade 3) toxicities—predominantly rash and diarrhea which cannot be controlled by medical management and supportive care measures, or major uncorrectable laboratory abnormalities—occur with cetuximab alone, subjects will not be eligible to receive the combination of VTX-2337 with cetuximab at Week 1. Specifically, subjects will be considered unevaluable and will be replaced.

In the absence of severe (≥ Grade 3) toxicities with cetuximab alone, subjects will proceed with treatment at Week 1 with combination cetuximab and VTX-2337. VTX-2337 will be

given once weekly for three weeks followed by a week off therapy in conjunction with cetuximab.

One cycle of therapy will consist of cetuximab administered IV weekly and VTX-2337 administered SC weekly for three consecutive weeks out of four starting at Week 1 (Cycle 1). The first cycle of therapy will end at the completion of Week 4. This first cycle of combination therapy will serve as the DLT assessment period.

Dose modifications, dose delays and other toxicities will be assessed throughout all courses of treatment when determining the optimal dose for phase II studies (RP2D). Refer to §5.10 for a detailed description of these processes.

4.8.1 Cetuximab Lead-In Period

For cetuximab naïve subjects, a loading dose of intravenous (IV) cetuximab at 400 mg/m^2 will be administered on Day -28 of Week -4, followed by IV cetuximab at 250 mg/m^2 given weekly on weeks -3, -2, and -1. For subjects previously treated with cetuximab, the lead-in period will consist only of one dose of cetuximab (a loading dose of intravenous (IV) cetuximab at will be administered at 400 mg/m^2 if greater than 3 weeks have elapsed since prior cetuximab therapy, otherwise a 250 mg/m^2 cetuximab dose will be administered) , administered on Week -1. An assessment of rash and toxicities due to cetuximab alone will be performed on or prior to initiating combined therapy in Cycle 1 Week 1. Toxicities identified during the Lead-In period will not be used towards the determination of DLT and subjects who develop severe toxicities during this period will be considered unevaluable, withdrawn from study protocol for combined therapy and replaced.

Patients who experience CTCAE rash or non-hematologic toxicity ≥ Grade 3 prior to Week 1 will be withdrawn from study and will not receive the investigational agent in combination.

4.8.2 Combination Therapy Initiation at Cycle 1

If rash or other non-hematologic toxicities attributable to cetuximab alone are ≤ Grade 2, weekly therapy with cetuximab will continue in Cycle 1. Subcutaneous (SC) injections of VTX-2337 will be initiated on Cycle 1 Day 1, and continued on a schedule of once weekly dosing for 3 consecutive weeks followed by a one week break (cycle Week 4). See also Appendix A and §5.3.

4.8.3 Duration of Treatment

The duration of treatment will depend on individual response and tolerance. Patients will receive subsequent treatment cycles until they meet the criteria for discontinuation or withdrawal from the study.This includes progressive disease (except as noted below), withdrawal of consent, and clinical judgment of the Investigator, intolerable toxicity, death, or Investigator-Sponsor discontinuation of the study.

Patients with clearly documented progressive disease will generally be taken off study at the time of progression. However, given the immunological mechanism of action of VTX 2337 and the potential for delayed evidence of clinical efficacy while immunologic responses develop, patients are allowed to remain on study for 1–2 cycles following documented disease progression at the discretion of the Investigator. Such patients should continue to be followed per protocol. After 1–2 additional cycles of treatment, if disease progression is again confirmed, the patient should be removed from the study, and treated as per the Investigator's discretion clinically.

Patients who are responding (partial response) or whose disease is stable will generally be treated until disease progression, intolerable toxicity or patient refusal to continue with study.

Refer also to §5.5.

Figure 1: Treatment Schema

Initial Study Enrollment (Lead-In period of 4 weeks prior to Cycle 1 start at Week 1. Each cycle of therapy is 4 weeks in duration. Cycle 2 starts Week 5).

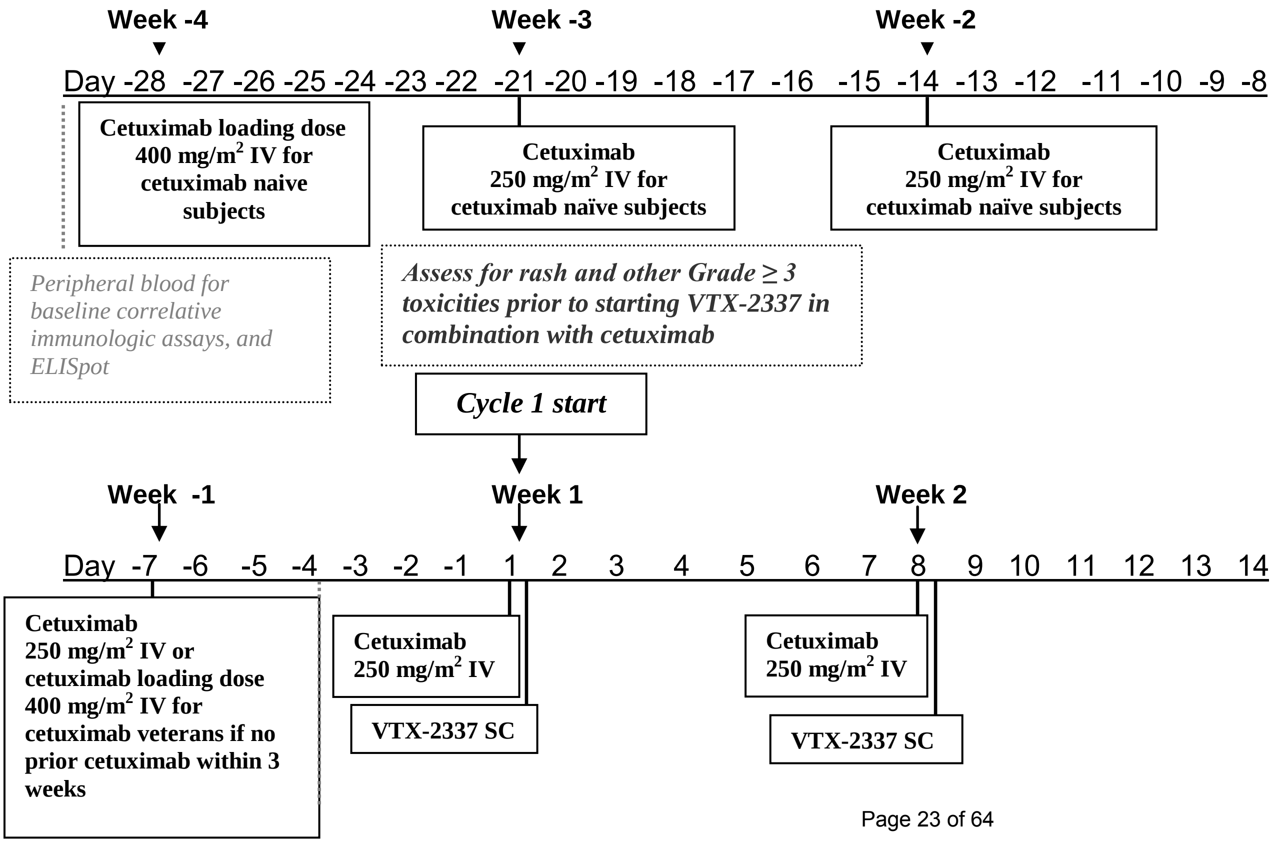

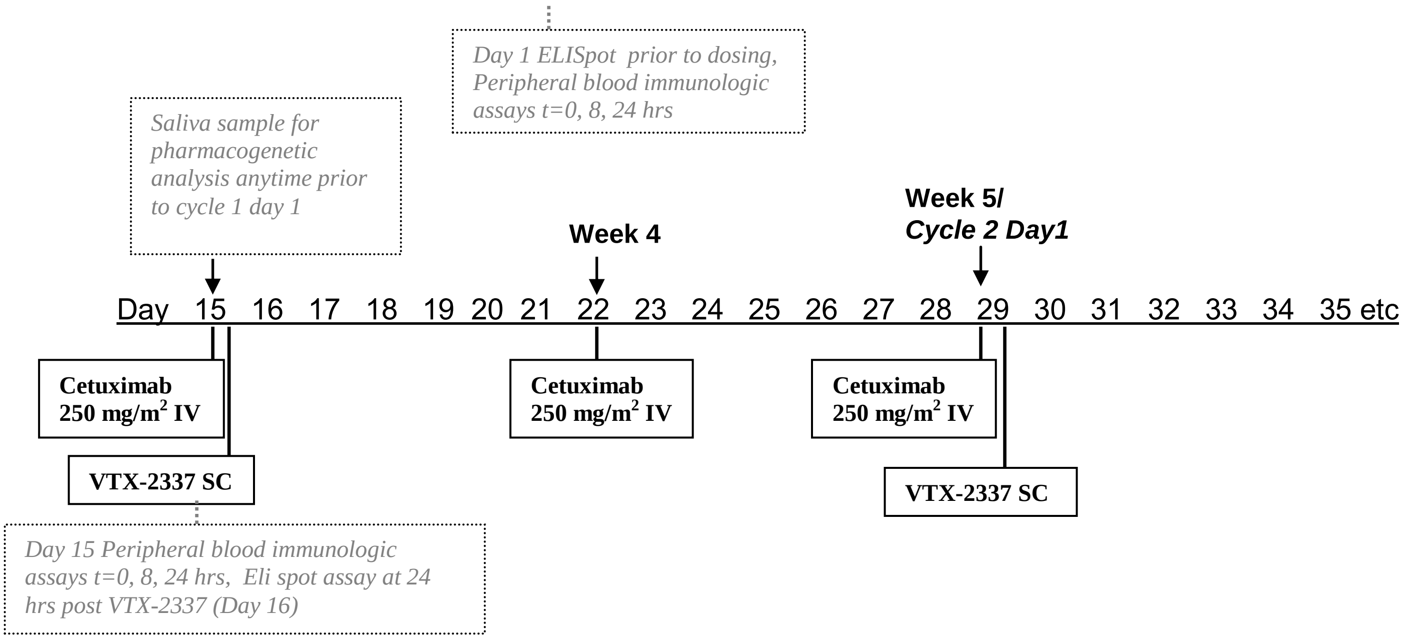

5.0 STUDY CONDUCT AND TRIAL PROCEDURES

Prior to undergoing any study-specific procedure, patients must read and sign the current Institutional Review Board (IRB)-approved informed consent form. All on-study procedures are permitted within the window frame indicated in Schedule of Protocol Activities (Appendix A).

5.1 Screening

The following screening procedures should be performed within 7 days prior to initiation of treatment on-study (cetuximab lead-in) unless otherwise specified:

- Patient signature on current IRB-approved informed consent form. May be obtained up to 30 days prior to treatment.
- Medical history and demographics.
- ECOG performance status, body weight, and vital signs (temperature, blood pressure, heart rate, respiratory rate).
- Physical examination, including examination of major body systems.
- Hematology and Chemistry as described in Appendix B.
- Urinalysis.
- Pregnancy test (serum or urine), if applicable. May be performed up to 21 days prior to treatment.
- Obtain peripheral blood samples for correlative research studies, ELISpot.
- 12-lead ECG.

- Tumor imaging, including CT, PET/CT or MRI scans of the chest and other known sites of disease.
  - May be done up to 28 days prior to treatment.
  - Note that the same imaging modality must be used for each patient throughout the study.
- Brain CT or MRI scan for known (treated) or suspected brain metastases. May be done up to 28 days prior to treatment. Imaging of the brain is not needed in the absence of clinical symptoms or clinical suspicion of brain metastases or CNS involvement.
- Bone scan for patients with known or suspected bone metastases. May be done up to 28 days prior to treatment. Bone scan not needed in the absence of clinical symptoms or lack of clinical suspicion for bone metastases.

The results of all screening assessments should be reviewed by the Investigator to ensure the patient meets all eligibility requirements as outlined in §3.0. Thereafter, qualified subjects can begin the cetuximab lead-in treatment.

5.2 Cetuximab Lead-In Period

Please refer to Appendix A for a comprehensive list of required assessments and procedures.

- As per standard of care, a cetuximab loading dose will be administered at 400 mg/m^2 IV. The loading dose will be given on Week -4, Day -28 for cetuximab naïve subjects, and Week -1 if no prior cetuximab within 3 weeks in previously treated subjects.
- For cetuximab naïve subjects, subsequent infusions at a dose of 250 mg/m^2 IV will be administered starting Week -3, Day -21 and continued through weekly for 3 doses. Subjects previously treated with cetuximab will receive the loading dose only prior to Reassessment.
- Weekly laboratory work may be performed as per local clinical practice. Hematology and chemistry as per Appendix B are suggested.

Reassessment (within 7 days of Cycle 1)

Prior to initiation of VTX-2337 in combination with cetuximab, the following criteria must be met:

- Subjects must have received a single dose of cetuximab (the loading dose of cetuximab at 400 mg/m^2 (if no prior cetuximab within 3 weeks in previously treated subjects) and, for cetuximab- naïve subjects, subsequently at least three once-weekly doses of cetuximab at 250 mg/m^2.
- Assessment of rash, all toxicities, and all drug-related adverse events.

Patients should not have any current or ongoing ≥ Grade 3 non-hematologic toxicities due to cetuximab alone.

Specifically, subjects with ≥ Grade 3 (severe) rash due to cetuximab prior to Week 1 will be discontinued from study, will not receive VTX-2337 and will be replaced.

- The subject must demonstrate adequate bone marrow, renal, and hepatic function—with corrected normal potassium and magnesium levels—as evidenced by the following:
  - Absolute neutrophil count (ANC) ≥ 1,500 cells/µL
  - Platelet count ≥ 75,000 cells/µL
  - Hemoglobin ≥ 8.0 g/dL
  - Creatinine ≤ 2.0 mg/dL
  - Total bilirubin ≤ 2.0 x ULN
  - SGOT(AST), SGPT (ALT) ≤ 2.5 x ULN

    (For patients with liver metastases, AST, ALT <5x ULN is acceptable)

Subjects, who cannot meet the minimal criteria above prior to starting Cycle 1 (Week 1) with combined cetuximab and VTX-2337, will be replaced.

5.3 Study Period (cetuximab + VTX-2337)

Refer to Appendix A for a comprehensive list of required assessments and procedures.

Refer to §5.7 for details on cohort enrollment, assignment of VTX-2337 dose level, and assessment of dose-limiting toxicities.

Cycle 1 Procedures Prior to Initiation Therapy

The following assessments must be performed on Day -1 or Day 1 of Cycle 1, prior to the doses of cetuximab and VTX-2337.

- Assessment of baseline signs and symptoms/adverse events. Note that cetuximab-related toxicities should be noted as part of the patient's baseline condition prior to initiation of combination therapy.
- ECOG performance status, body weight, and vital signs.
- Physical examination including major body systems.
- Hematology and Blood Chemistry (see Appendix B) if not performed within the previous 7 days.
- Saliva samples for pharmacogenetic evaluation can be performed anytime prior to initiation of combined therapy.

The following assessment must be performed on Cycle 1 Day 1 prior to the doses of cetuximab and VTX-2337:

- Blood samples for immune monitoring and correlative research studies.

Cycle 1 (Weeks 1 to 4) Treatment Procedures

A cycle of therapy will start from Week 1 Day 1 to the end of Week 4 Day 28.

The following procedures must be performed during Cycle 1:

- Treatment with cetuximab on Day 1, 8, 15 and 22.
- Treatment with VTX-2337 on Day 1, 8 and 15.
- Assessment of adverse events and tumor-related signs and symptoms at each study visit.
- Hematology and blood Chemistry (including electrolytes and magnesium; see Appendix B) according to the schedule in Appendix A.
- Correlative studies: peripheral blood for immunologic assays (Rules Based Medicine immune monitoring and flow cytometry) should be obtained as follows:
    - Cycle 1 Day 1 prior to dosing of cetuximab or VTX-2337 (“pre-dose”; time 0h), and then subsequently at 8 hours and 24 hours (Day 2) post VTX-2337 dosing.
    - Cycle 1 Day 1 ELISpot will be performed prior to dosing with cetuximab.
    - Cycle 1 Day 15 prior to dosing (pre-dose; time 0h), and 8 hrs and 24 hrs (Day 16) post VTX-2337 administration.
    - Cycle 1 Day 16, ELISpot to be performed 24 hours after VTX-2337 administration.

Cycle 2 (Weeks 5 to 8) and Subsequent Cycles

The following procedures must be performed during Cycle 2 and subsequent cycles of therapy:

- Assessment of adverse events and tumor-related signs and symptoms at each study visit.

  Starting with Cycle 3, only adverse events that can reasonably be regarded as caused by the study drug (VTX-2337) should be captured throughout the remainder of the study.
- ECOG performance status, body weight, and vital signs on Day -1 or Day 1 of each cycle.

- Physical examination, including major body systems, on Day -1 or Day 1 (all cycles). A problem-oriented PE can be conducted at the Investigator's discretion at any time.
- Hematology and blood Chemistry (including electrolytes and magnesium; see Appendix B) according to the schedule in Appendix A.
- Treatment with cetuximab on Days 1, 8, 15, and 22 of each 28-day cycle. (Flexibility of +/- 1 day for subject convenience and holidays without constituting a protocol deviation)
- Treatment with VTX-2337 on Days 1, 8 and 15 of each 28-day cycle. [No treatment on Day 22]. (Flexibility of +/- 1 day for subject convenience and holidays without constituting a protocol deviation)
- Tumor imaging, including CT or MRI scans of the chest and other applicable sites of disease (must be the same imaging modality(ies) and anatomical sites as Screening assessments) at the end of every second cycle. E.g. end of Cycle 2 (prior to starting Cycle 3), end of Cycle 4, end of Cycle 6, etc.

Treatment criteria and Adjustments for Toxicity:

Adequate parameters of Hemoglobin (>8.0 g/dL), Platelets (>75 x 10^9/L or >75,000/mm^3), ANC (>1.5), and non-hematologic toxicity ≤ Grade 2 will be the minimal criteria to proceed with weekly cetuximab and/or VTX-2337 therapy.

Should these parameters not be met, cetuximab and/or VTX-2337 infusion should be held and delayed until hematologic parameters are met or non-hematologic toxicity resolves to baseline or ≤ Grade 2.

Refer also to §5.10.

5.4 Concomitant Medications and Supportive Care

The following medications and interventions are prohibited from the time of study screening until the End of Study visit:

- Systemic anti-cancer therapy (other than cetuximab), including chemotherapy, immunotherapy, or hormonal cancer therapy or experimental therapy.
- Systemic – oral, intravenous, and injectable - corticosteroids (e.g., dexamethasone) should be avoided except for symptom management and supportive care, and should be limited to brief courses of therapy (< 10 days) at the lowest doses possible.
- Any investigational device or drug other than VTX-2337

Subjects should be provided with full supportive care measures as clinically indicated, and in accordance with institutional standards. Such care includes medication for pain control and symptom management, antibiotics, bisphosphonates, antiemetics, colony stimulating factors, and transfusions of blood or blood products.

Localized radiotherapy is permitted for palliation of painful lesions at the Investigator's discretion.

5.5 End of Study Treatment / Withdrawal Procedures

Patients will have completed their participation in the study in the case of:

- Disease progression, except as described below
- Unacceptable toxicity
- Need for treatment rest > 21 days; refer to §5.10
- Need to reduce dose of VTX-2337 more than 2 dose levels; refer to §5.10
- Need for anticancer therapy not specified in the protocol
- Patient noncompliance
- Patient lost to follow-up
- Patient choice to withdraw from treatment (follow-up permitted by patient; see below)
- Withdrawal of patient consent (cessation of follow-up; see below)
- Study closure by Sponsor-Investigator

Patients with clearly documented progressive disease will generally be taken off study at the time of progression. However, given the immunological mechanism of action of VTX 2337 and the potential for delayed evidence of clinical efficacy while immunologic responses develop, patients are allowed to remain on study for 1 to 2 cycles following documented disease progression at the discretion of the Investigator. Such patients (e.g. those for whom there is reasonable evidence of clinical benefit to justify continuation on protocol) should continue to be followed per protocol. After 1 to 2 additional cycles of treatment, if disease progression is again confirmed, the patient should be removed from the study.

Subjects may withdraw from the trial at any time at their own request, or they may be withdrawn at any time at the discretion of the Investigator or sponsor for safety, behavioral, or administrative reasons.

If the subject withdraws from the trial and also withdraws consent for disclosure of future information, no further evaluations should be performed and no additional data should be collected. The sponsor may retain and continue to use any data collected before such withdrawal of consent.

At the end of the study or at withdrawal, the following procedures should be performed if they were not performed during the last week on study:

- ECOG performance status, body weight, and vital signs

- Assessment of adverse events and tumor-related signs and symptoms
- Physical examination including major body systems
- Hematology and Blood Chemistry as described in Appendix B
- Tumor imaging including PET/CT, CT or MRI scans of known sites of disease (utilizing the same imaging modalities and anatomical areas as Screening)

5.6 Follow-up Visit Procedures

Patients should continue to be evaluated for 28 calendar days after the last dose of study treatment. At the post-treatment follow-up visit, the following procedures should be performed:

- Assessment of adverse events and tumor-related signs and symptoms.
- Physical examination, ECOG performance status, body weight, vital signs, laboratory assessments, or other tests necessary to follow unresolved adverse events.

During this period, the outcome of adverse events with a date of onset during the study period should be reevaluated. Adverse events will be followed until they are resolved or until a new anti-cancer treatment is initiated. All serious adverse events, and those non-serious adverse events assessed by the Investigator as possibly related to study drug, should continue to be followed even after patient withdrawal from study. These adverse events should be followed until they resolve or until the Investigator assesses them to be “chronic” or “stable.”

5.7 Cohort Enrollment, Expansion & Replacement of Patients

This study will evaluate cetuximab in combination with VTX-2337 in a dose-escalation scheme, whereby cohorts of n=3 patients will be enrolled and administered increasing doses of the investigational drug until the MTD/RP2D is determined.

For each cohort, following the lead in period with cetuximab, 3 patients will be enrolled and administered one cycle of cetuximab plus VTX-2337: “Cycle 1”; cetuximab 250 m/mg^2 on Days 1, 8, 15 and 22 plus VTX-2337 at the assigned dose level on Days 1, 8, and 15. Safety data through Cycle 1 for all 3 patients must be reviewed before enrollment of the next cohort can be considered.

If none of the 3 patients experiences a DLT (see §5.8) during Cycle 1, dose escalation may proceed and 3 new patients may be enrolled in the next cohort (cohort 2) and administered cetuximab plus VTX-2337 at the next dose level. Cohort 3 will be enrolled and advanced in this same manner.

If 1 of 3 patients in a cohort experiences a DLT during Cycle 1, the cohort will be expanded and up to 3 additional patients must be treated at that dose level before VTX-2337 dose

escalation can proceed. If a total of 1 of 6 patients experiences a DLT during Cycle 1, dose escalation may continue. If 2 or more patients in a cohort experience a DLT in Cycle 1, no additional patients will be treated at that dose of VTX-2337, and that dose level will be considered unacceptable. Once the MTD/RP2D has been identified, up to 3 additional patients should be enrolled at that dose level of VTX-2337 to ensure a total of 6 patients are treated at the RP2D.

Patients will be initiated at Dose Level 1. In the case of ≥ 2 patients with DLT, a de-escalation will occur to enroll patients onto Dose Level -1.

5.8 Dose-Limiting Toxicities (DLT)

Toxicity will be evaluated using the National Cancer Institute Common Terminology Criteria for Adverse Events (NCI CTCAE v 4.0).

Dose Limiting Toxicity (DLT) is defined as any study-drug related adverse event, occurring during Cycle 1 (Week 1 through Week 4), which meets the following criteria:

- Any Grade ≥ 4 hematologic toxicity.
- Any ≥ Grade 3 non-hematologic toxicity, with the exception of:
  - Grade 3 hypersensitivity reaction.
  - Grade 3 localized injection-site toxicities.
- Diarrhea, nausea or vomiting will only be considered dose limiting toxicity when ≥ Grade 3 toxicity occurs despite adequate anti-emetics or anti-diarrhea medications.
- Treatment delay due to toxicity lasting greater than 21 days since the last dose of cetuximab or VTX-2337.
- Uveitis
- Death

"Study-drug related" refers to events that are possibly or probably related to the administration of VTX-2337 or the combined treatment regimen of VTX-2337 and cetuximab, and not clearly attributed to cetuximab alone or other cause. (Toxicities during the Lead-In Period (Weeks - 4 to - 1) will not be considered a DLT as the study drug VTX-2337 will not have been administered in combination with cetuximab.)

Patients will be replaced if:

- they are unable to complete dosing in Cycle 1.
  OR
- they must discontinue the study prematurely due to disease-related complications (e.g., disease progression) or for any other reason(s).
  AND
- they have not experienced a DLT

Note: DLTs are adverse events that occur during the administration of Cycle 1 and meet the specific criteria outlined above. The presence or absence of DLTs dictates whether cohorts must be expanded and whether or not dose escalation (enrollment of the next cohort) can occur. However, individual subjects who experience DLTs may nevertheless receive additional doses of VTX-2337, following careful medical evaluation and appropriate treatment and/or a dose delay of 1 week, at the discretion of the Investigator.

5.9 Maximum Tolerated Dose

The maximum tolerated dose (MTD) will be the highest dose at which no more than one of six patients experience a DLT. Alternatively, the MTD may be determined in conjunction with VentiRx Pharmaceuticals based on emerging data from other concurrent Phase 1 trials. The MTD level in this study will be expanded to enroll up to 6 evaluable patients. The MTD and general tolerability will be used in the determination of the RP2D.

5.10 Dose Modifications

In order to maintain dose-intensity and cumulative dose-delivery on this study, reasonable efforts will be made to minimize dose reduction and treatment delays as specified. No intra-patient dose escalation is planned for this study.

Patients should be monitored for study-drug related toxicity according to the procedures outlined in Appendix A.

The investigator should carefully assess all treatment-associated toxicities and, whenever possible, determine if they can reasonably be attributed to cetuximab alone, VTX-2337 alone, or the combination regimen. If appropriate, dose delays and/or adjustments should be restricted to the suspected causative agent.

- As previously noted, toxicity grades are defined using the NCI CTCAE version 4.0.
- If therapy for either study drug is delayed due to drug-related toxicities, dosing with the other (non-toxic) drug may proceed without interruption if, in the Investigator's opinion, the event can reasonably be attributed to a single causative agent and dosing with the other drug is not contraindicated.

  If drug-related toxicities cannot reasonable be attributed to only one of the study drugs, both drugs will be held until recovery to ≤ Grade 2.

- In general, no more than two dose reductions are allowed for hematological or non-hematological toxicities of either drug (i.e. any patient who has had 2 dose reductions and who experiences a toxicity that would cause a third dose reduction should be discontinued from the study and treated at the investigator's discretion).
- Any patient who requires a dose reduction of cetuximab or VTX-2337 will continue to receive the reduced dose for the remainder of the study.

- No dose escalations are allowed in this study.

5.10.1 Cetuximab Dose Modifications

Refer to the cetuximab (Erbitux) label for current recommendations regarding dose delays and reductions.

In case of delayed cetuximab treatment, there will be no new 400 mg/m^2 initial dose at the restart of treatment, and all subsequent treatments will be given at the same dose level as the last administered dose, unless further dose delays or reductions are needed. Once the dose of cetuximab has been reduced due to drug-related toxicity, it should not be increased at a later time.

If cetuximab therapy is delayed due to drug-related toxicities, dosing with VTX-2337 may proceed without interruption if, in the Investigator's opinion, the event can reasonably be attributed to cetuximab alone and VTX-2337 dosing is not contraindicated. If cetuximab is permanently discontinued for any reason, VTX-2337 will not be administered alone and the patient will be discontinued from the study.

Dose Modifications for Infusion Reaction

- Serious infusion reactions occurred with the administration of cetuximab in approximately 3% of patients in clinical trials.[38]
- Reduce the infusion rate by 50% for NCI CTCAE Grade 1 or 2 and non-serious NCI CTCAE Grades 3–4 infusion reactions.
- Immediately and permanently discontinue cetuximab for serious infusion reactions, requiring medical intervention and/or hospitalization.

In each case of an allergic/hypersensitivity reaction, the Investigator should institute treatment measures according to local practice. Based on previous experience with cetuximab allergic/hypersensitivity reactions, the following treatment guidelines are applicable:

Treatment Guidelines for Cetuximab Hypersensitivity Reactions

CTCAE Grade Allergic/Hypersensitivity Reaction	Treatment
**Grade 1**<br>(transient rash, drug fever <38°C)	• Decrease the cetuximab infusion rate by 50% and monitor closely for any worsening.<br>• The total infusion time for cetuximab should not exceed 4 hours (400 mg/m^2) or 2 hours (250 mg/m^2), respectively.
**Grade 2**<br>(Urticaria, drug fever ≥ 38°C and/or asymptomatic bronchospasm)	• Stop cetuximab infusion.<br>• Resume infusion at 50% of previous rate once allergic/hypersensitivity reaction has resolved or decreased to Grade 1 in severity, and monitor closely for any worsening.
**Grade 3 or Grade 4**<br>(Grade 3: Symptomatic bronchospasm requiring parenteral medication, with or without urticaria; hypersensitivity-related edema, angioedema.<br>Grade 4: Anaphylaxis)	• Stop the cetuximab infusion immediately and disconnect infusion tubing from the patient.<br>• Patients have to be withdrawn immediately from the treatment and must not receive any further cetuximab treatment.

Dose Modifications for Dermatologic Toxicity

Patients should be assessed for rash carefully throughout the study; assessment should include clinical examination and—optionally—photographic imaging, if beneficial.

- Acneiform rash occurs in up to 88% of patients receiving cetuximab. Severe acneiform rash occurs in up to 17% of patients.[38]

- Acneiform rash usually developed within the first two weeks of therapy. Monitor patients receiving cetuximab for dermatologic toxicities and infectious sequelae.

- If a patient experiences a Grade 3 skin reaction, the Investigator should consider concomitant treatment with topical and/or oral antibiotics; topical corticosteroids are not recommended. If the toxicity resolves to Grade 2 or less within 2 weeks, treatment may resume.

- Skin toxicities can be managed as per Figure 2. Recommended dose modifications for severe (Grade 3 or 4) acneiform rash are specified below.

Patients will be removed from the study—and treated at the Investigator's discretion—in event for the following:

- Discontinuation of therapy for more than 2 consecutive infusions of cetuximab (21 days without therapy)
- Grade 4 skin reaction.

Figure 2: Treatment Algorithm in Case of Cetuximab-related Skin Toxicity

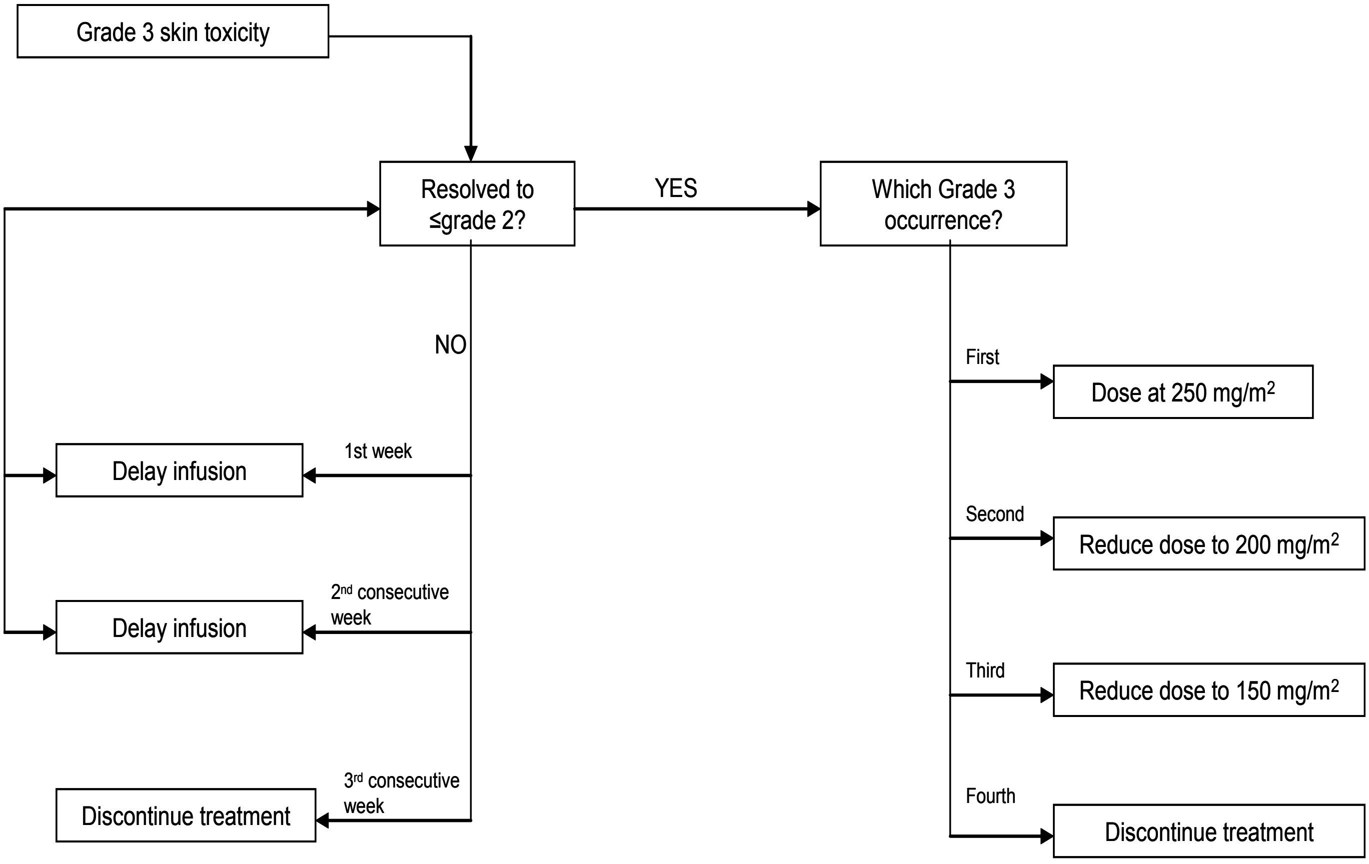

If a patient experiences a Grade 4 skin reaction, cetuximab therapy will be discontinued.

Dose Modifications for Pulmonary Toxicity

If interstitial lung disease is confirmed, cetuximab should be discontinued and the patient should be treated appropriately.

Dose Modifications for Other Reasons

If a patient develops an intercurrent illness (e.g., infection) that, in the opinion of the Investigator mandates interruption of therapy, this intercurrent illness must resolve within a time frame such that no more than 2 consecutive cetuximab infusions are withheld. If cetuximab must be withheld for a longer period of time, cetuximab treatment will be discontinued, with the exception of a patient who is benefiting from cetuximab treatment.

5.10.2 VTX-2337 Dose Modification

VTX-2337 is a non-cytotoxic therapy. In previous clinical and non-clinical studies of this agent, no drug-related laboratory toxicities have been observed (i.e. no hematological toxicity). In addition, most observed drug-related adverse events, such as flu-like symptoms and fever, resolve in < 48 hours and do not require dose reductions or a delay in the weekly dosing regimen. As a result, the most common adjustment to VTX-2337 dosing is a delay of 1–2 weeks to allow recovery from localized injection-site reaction. A dose reduction is permissible for drug-related adverse events at the treating investigator's discretion.

Accordingly, doses of VTX-2337 may be delayed for up to two weeks at any time if warranted due to drug-related adverse events. Patients who require (1) a dose delay of > 2 weeks or (2) require more than 2 such delays at a given dose level should have VTX-2337 reduced by 1 dose level. Once the dose of VTX-2337 has been reduced due to drug-related toxicities, it should not be increased at a later time. Once the MTD/RP2D has been determined, all active patients receiving a higher dose of VTX-2337 will be subsequently treated at the MTD/RP2D.

Patients who require > 2 dose reductions of VTX-2337 due to drug-related toxicity should be discontinued from the study.

Guidelines for treating ocular toxicities are provided in Appendix G for consideration, and have been developed in consultation with ophthalmologic experts.

5.11 Correlative Research (Immunologic Response)

VTX-2337 has been shown to induce biomarkers consistent with the activation of both innate and adaptive immunity in vitro and in vivo. Immunologic biomarkers proposed (see below) will assess markers representative of both arms of the immune system.

Additionally, genetic variants (single-nucleotide polymorphisms, SNPs) have been identified in both the TLR8 gene and Fc gamma-III gene which could potentially impact

response to treatment with VTX-2337. Patient samples will undergo genotyping assays to allow genetic characterization of study participants.

5.11.1 Assessment of Baseline Immune Status

Baseline immune responses will be evaluated in vitro in all patients to explore the possibility that differences in immune responsiveness will correlate with clinical efficacy and/or safety. Blood samples will be obtained prior to the cetuximab run-in and evaluated using protein array technology (Rules Based Medicine, TruCulture™). The TruCulture closed-culture system provides a quantitative assessment of cytokine and chemokine responses to validated immunostimulatory agents.

Prior to initiating run-in with cetuximab, draw 1 mL of blood directly into the TruCulture tube using the provided kits. Place the culture tube immediately in a dry heat block at 37°C for 24–48 hours. After culture, follow the provided instructions to manually insert the valve into the culture tube to separate the supernatant from the cells. Using appropriate technique, transfer the plasma into an appropriately-labeled tube. **Freeze immediately at -70ºC until shipment.**

Frozen samples, including a sample manifest, should be stored until completion of study accrual. They should subsequently be shipped in an insulated container with dry ice to:

Rules-Based Medicine, Inc.
3300 Duval Road
Austin, TX 78759
Tel: 512.835.8026
Fax: 512.835.4687

Shipments must be marked for overnight delivery, and can be shipped on Monday through Thursday.

5.11.2 Assessment of Cytokine Response

Studies in non-human primates and in cancer patients have shown that specific cytokines, chemokines and other inflammatory markers are elevated in the peripheral blood of after administration of VTX-2337. Samples for immunologic monitoring (quantitative assessment for specific analytes by protein array technology; Rules Based Medicine HuMAP) will be obtained for each patient according to the schedule in Appendix A.

At the time points specified in Appendix A ("Immune Monitoring"), draw at least 3 mL of blood into a lavender-top K2 EDTA tube using standard venipuncture techniques. Invert the filled tube gently at least 8 to10 times. [One complete inversion is to turn the filled tube upside-down and return it to the upright position.] Centrifuge the blood sample within 1 hour of collection time at 1500 x g for 15 minutes at 2ºC–8ºC until cells and plasma are well separated. Use a sterile pipette, transfer all the plasma into an appropriately-labeled tube. **Freeze immediately at -70ºC until shipment.**

Frozen samples, including a sample manifest, should be stored until completion of study accrual. They should subsequently be shipped in an insulated container with dry ice to:

Rules-Based Medicine, Inc.
3300 Duval Road
Austin, TX 78759
Tel: 512.835.8026
Fax: 512.835.4687

Shipments must be marked for overnight delivery, and can be shipped on Monday through Thursday.

5.11.3 Assessment of NK Cells

VTX-2337 has been shown to be a potent mediator of ADCC, effectively increasing target lysis in in-vitro culture. Assays will be performed (University of Washington Tumor Vaccine Group) to evaluate the activation and function of NK cells after administration of VTX-2337. Samples for assessment of NK cells via flow cytometry will be obtained for each patient according to the schedule in Appendix A.

At the time points specified in Appendix A ("Flow Cytometry"), draw at least 10 mL of blood into each of two green-top heparin tubes (20 mL total) using standard venipuncture techniques. Invert the filled tube gently at least 8–10 times. [One complete inversion is to turn the filled tube upside-down and return it to the upright position.]

Tubes should be appropriately labeled and transported via same-day courier to:

University of Washington, Tumor Vaccine Group
Attn: Yushe Dang
815 Mercer Street, Room 219
Seattle, Washington 98109-8050
Telephone: (206) 616-8448

Upon receipt of samples at the TVG, CD56 cells will be stained and analyzed by flow cytometry for surface markers: CD69 (activation) and intracellular stains: IFN-gamma and CD107 (function-cytokine secretion and degranulation). Separate reactions will be performed for IFN-gamma and CD107 (i.e. each staining will include 2 surfaces and 1 intracellular stain).

5.11.4 IFN-gamma ELISPOT:

VTX-2337 has been shown to activate IL-12 secreting dendritic cell populations in human PBMC. Consequently, administration of VTX-2337 may stimulate adaptive immune responses. Several immunogenic proteins—EGFR and CD105—have been identified as biologically relevant antigens for head and neck cancer. In multivariate analyses, these proteins have been found to be indicators of poor clinical outcome when they are overexpressed in head and neck tumors. Moreover, these proteins have been shown to be human tumor antigens by the UW Tumor Vaccine Group and others. Antigen-specific IFN-gamma secretion has been shown to be correlated to beneficial clinical outcome after

immunotherapy. Preliminary information as to the ability to stimulate such responses after administration of VTX-2337 will be collected. Samples for assessment by ELISpot will be obtained for each patient according to the schedule in Appendix A.

At the time points specified in Appendix A ("ELISPOT"), draw at least 10 mL of blood into each of eight green-top heparin tubes (80 mL total) using standard venipuncture techniques. Invert the filled tube gently at least 8–10 times. [One complete inversion is to turn the filled tube upside-down and return it to the upright position.]

Tubes should be appropriately labeled and transported via same-day courier to:

University of Washington, Tumor Vaccine Group
Attn: Yushe Dang
815 Mercer Street, Room 219
Seattle, Washington 98109-8050
Telephone: (206) 616-8448

Upon receipt of samples at the TVG, PBMC will be purified from peripheral blood and plated at $1x10^5$/well in quadruplicate for each antigen. A 10-day ELISpot will be performed in accordance to the methods described by Disis et. al.[39] All samples for individual patients will be cryopreserved then reconstituted and analyzed at the same time at the end of study to correct for variation. Antigens to be tested include:

- No antigens (media only)
- HIV peptide (negative antigen control)
- recombinant proteins for EGFR and CD105
- CEF peptide pool (positive antigen control)
- PHA

5.11.5 Genetic Analysis

Functional genetic variants (single-nucleotide polymorphisms; SNPs) have been identified in both the TLR8 gene (the A1G allele) and Fcγ Receptor IIIA gene (158 F allele) in humans. Functional SNPs could potentially impact response to treatment with VTX-2337. To assess whether patients with SNPs have altered biological and/or clinical responses to VTX-2337 as compared to patients without such polymorphisms, a sample will be obtained from all patients enrolled in the study for pharmacogenomic analysis.

A saliva sample will be collected from each eligible subject prior to the Cycle 1 Day 1 dose of VTX-2337. Samples should be appropriately labeled and transported to:

University of Washington, Tumor Vaccine Group
Attention: Yi Yang
815 Mercer Street, Room 219
Seattle, Washington 98109
Telephone: (206) 616-8448

Upon receipt of samples at the TVG, pharmacogenomic analysis will be performed to TLR8 and FcγR characterize SNPs in the IIIA genes by conventional methods.

6.0 DATA ANALYSIS/STATISTICAL METHODS

Sample Size Determination: The number of patients to be enrolled in the study will depend upon the observed safety profile, which will determine the number of patients per dose level and the number of dose escalations. It is anticipated that a total of approximately 12 to 15 patients (maximum of 18 patients) will be enrolled in this study with an estimated annual accrual of 12. The study will take place at a single center.

The operating characteristics of this study design are shown in Table 1, which provides the probability of escalation to the next higher dose for each underlying true DLT rate. For example, for a toxicity that occurs in 5% of subjects, there is a greater than 95% probability of escalating. Conversely, for a common toxicity that occurs with a rate of 70%, the probability of escalating is <5%.

Table 1: Probability of Escalation to the Next Dose for Each True Underlying DLT Rate at a Dose Level

True Underlying DLT Rate	5%	10%	20%	30%	40%	50%	60 %	70%	80%	90%
Probability of Escalating Dose	0.97	0.91	0.71	0.49	0.31	0.17	0.0 8	0.03	0.01	0.00 1

Table 2 shows the probability of failing to observe toxicity in a sample size of 3 or 6 patients given various true underlying toxicity rates. For example, with 6 patients, the probability of failing to observe toxicity occurring at least 40% of the time is less than 5%.

Table 2: Probability of Failing to Observe True Underlying DLT Rate at a Dose Level

True Underlying DLT Rate	5%	10 %	20%	30 %	40%	50%	60%	70%	80%	90%
Probability of Failing to Observe Toxicity, N=3	0.8 6	0.7 3	0.51	0.3 4	0.22	0.13	0.064	0.027	0.008	0.00 1
Probability of Failing to Observe Toxicity, N=6	0.7 4	0.5 3	0.26	0.1 2	0.04 7	0.01 6	0.004 1	<0.00 1	<0.00 1	<0.0 01

Data Analysis

The study population for toxicity analyses will include all patients enrolled in the study who receive at least one dose of study medication VTX-2337. Efficacy will be determined based on patients completing at least to the end of Cycle 1. Due to the exploratory nature of this study, no confirmatory inferential analyses are planned, and no imputation for missing data will be done. Descriptive statistics (such as means, medians, standard deviations and ranges for continuous data and percentages for categorical data) will be used to summarize patient characteristics, treatment administration/compliance, efficacy,

safety, and pharmacodynamic and correlative study data. Data will also be displayed graphically, where appropriate.

6.1 Analysis of Primary Endpoint

Primary endpoint: to determine the maximum tolerated dose (MTD)/RP2D and to define the toxicities of VTX-2337 in combination with cetuximab. For each cohort DLT's will be summarized by category (hematologic and non-hematologic) and by MedDRA preferred term.

6.2 Analysis of Secondary Endpoints

Secondary endpoints include the analysis of biologic correlative assays and studies and will be predominantly descriptive with graphical information where available.

6.3 Other Endpoints

Other assessments include response and determination of progression-free survival (PFS) duration.

Objective Response:

For each cohort and tumor type, the best response (CR, PR, SD or PD according to RECIST criteria) for each patient with measurable disease who received at least one dose of VTX-2337 study medication will be listed.

Progression-free survival will be determined in days or week and waterfall plots and graphical data will be provided where suitable.

Analysis of Other Endpoints

Analysis of Clinical Labs: Listing tables will be prepared for each laboratory measure, and will be structured to permit review of the data by patient as they progress on treatment. The tables will list the schedule, day and cycle of treatment, VTX-2337 dose, cetuximab dose and associated NCI CTCAE toxicity grade.

Summary tables and graphic displays, as appropriate, will be prepared to examine the distribution of these toxicities per cycle.

Graphic displays and shift tables may be provided to illustrate the results over time on study. Assessment of cumulative toxicities may be made.

Interim Analysis

No interim analysis is planned.

7.0 ASSESSMENTS

Safety Assessment and Determination of Dose Level for Subsequent Patient Cohorts

7.1 Laboratory Safety Assessments

Comprehensive blood work: Hematology and blood chemistry will be drawn at the time points described in the Schedule of Events (Appendix A) and analyzed at local laboratories. Investigators may have additional blood tests performed for the purpose of planning treatment administration, dose modification, or following adverse events.

Pregnancy test: Serum or urine pregnancy test for women of childbearing potential will be performed by a local laboratory.

7.2 Other Safety Assessments

Physical examination[1]: at Screening, a complete medical history and physical examination—including vital signs, performance status - will be performed. This will be repeated at subsequent visits, according to Appendix A, to assess clinically significant changes.

7.3 Efficacy Assessment

Correlative Studies: Immunologic (blood) and pharmacogenetic (saliva) samples will be obtained and evaluated as described in §5.11.

Imaging: Screening radiographs (e.g. CT, CT/PET, MRI, bone scan) will be performed to assess patient eligibility and for baseline disease assessment. Thereafter, radiologic studies will occur at the end of two cycles of combination therapy (prior to initiating Cycle 3). Subsequent assessments for response will occur after every subsequent two cycles (8 weeks) of therapy (i.e. end of Cycle 4, Cycle 6, Cycle 8, etc.)

7.4 Adverse Events

Assessment of adverse events will include type, incidence, severity (graded by the National Cancer Institute [NCI] Common Terminology Criteria for Adverse Events [CTCAE], Version 4.0), duration, seriousness, and relatedness; and clinically significant laboratory abnormalities.

Patients who receive 1 or more doses of VTX-2337 will be evaluable for toxicity.

---

[1] Includes: general appearance, skin, neck, eyes, ears, nose, throat, breast, lungs, heart, abdomen, back, lymph nodes, extremities and nervous system. The physical examination will include examination of known and suspected sites of disease. Height will be recorded at baseline only. Body weight will also be recorded at the start of each cycle.

Refer to §7.5 for requirements related to Serious Adverse Events.

7.4.1 Adverse Event Reporting

In the event of an adverse event the first concern will be for the safety of the subject. The adverse event recording period will start on the first day of study treatment and end 30 days (+/- 3 days) of termination of VTX-2337 dosing. If a subject begins a new anticancer therapy, the adverse event reporting period for new non-serious adverse events ends at the time the new treatment is started.

All adverse events will be reported for the first 2 cycles of study drug. Thereafter, only AEs which can reasonably be attributed to VTX-2337 will be reported.

7.4.2 Adverse Event Causality

The Investigator will use the following definitions to assess the relationship of the adverse event to the use of study drug:

Related - An adverse event has a strong temporal relationship to study drug, recurs on re-challenge or is known to be an effect of the study drug. Another reasonable etiology either doesn't exist or is unlikely.

Possibly Related - An adverse event has a strong temporal relationship to the study drug and an alternative etiology is either equally or less likely when compared to the potential relationship to study drug.

Not Related - An adverse event is due to an underlying or concurrent illness or effect of another drug and is not related to the study drug (e.g., has little or no temporal relationship to study drug or has a much more likely alternative etiology).

7.5 Serious Adverse Event Reporting

Investigators are required to report to the Sponsor-Investigator ANY serious adverse event (SAE) as soon as possible.

An SAE is any sign, symptom or medical condition that emerges during treatment or during a post-treatment follow-up period that (1) was not present at the start of treatment and it is not a chronic condition that is part of the patient's medical history, OR (2) was present at the start of treatment or as part of the patient's medical history but worsened in severity and/or frequency during therapy, AND that meets any of the following regulatory serious criteria:

- Results in death
- Is life-threatening
- Requires or prolongs inpatient hospitalization
- Is disabling
- Is a congenital anomaly/birth defect
- Is medically significant or requires medical or surgical intervention to prevent one of the outcomes listed above.

Serious adverse events require immediate notification to the following party below, beginning from the time of the first dose of investigational product (VTX-2337) through and including 30 calendar days after the last administration of VTX-2337. All emergent SAEs should be recorded on a MedWatch 3500A Form and communicated to:

Laura QM Chow, MD
Division of Medical Oncology, Box 358081
University of Washington (SCCA)
825 Eastlake Avenue E, MS:G4-940
Seattle WA, 98109-1023
Fax 206-288-1435, phone 206-288-6968
lchow@seattlecca.org

SAEs that are related, unexpected and occur at the University of Washington/Seattle Cancer Care Alliance will be reported to the Cancer Consortium IRB within 24 hours of learning of the event. Events that are unrelated or expected will be reported to Consortium IRB in the annual renewal.

All SAEs that are unexpected (i.e., not in the current VTX-2337 Investigator Brochure) and considered related or possibly related to the use of the study drug must be reported to FDA within 15 calendar days, or within 7 calendar days if the SAE was fatal or life-threatening.

As the manufacturer of the investigational drug product, VentiRx is to be notified of all drug-related SAEs (expected or unexpected) within 24 to 48 hours.

8.0 REGULATORY, QUALITY AND ADMINISTRATIVE REQUIREMENTS

8.1 Source Data Verification

Annual monitoring of source documents will occur during the active treatment phase of the trial. Monitoring visits will be arranged by the study coordinator in conjunction with the Monitoring Program Coordinator at the Fred Hutchinson Cancer Research Center. The monitors are independent contractors and are external to the Cancer Consortium (University of Washington and Fred Hutchinson Cancer Research Center).

Study monitors will perform ongoing source data verification to confirm that critical protocol data transcribed on the CRFs by authorized site personnel are accurate, complete, and verifiable from source documents.

Source documents are where patient data are recorded and documented for the first time. They include, but are not limited to, hospital records, clinical and office charts, laboratory notes, memoranda, pharmacy dispensing records, recorded data from automated instruments, copies of transcriptions that are certified after verification as being accurate and complete, X-rays, patient files, and records kept at the pharmacy, laboratories, and medico-technical departments involved in a clinical trial.

Source documents that are required to verify the validity and completeness of data transcribed on the CRFs must never be obliterated or destroyed.

To facilitate source documentation verification, the investigator(s) and institution(s) must provide the Monitor direct access to applicable source documents and reports for trial-related monitoring, audits, and IRB/EC review.

The investigational site must also allow inspection by applicable regulatory authorities.

8.2 Compliance with Laws and Regulations

The proposed study will be conducted according to the International Conference on Harmonization E6 Guideline for Good Clinical Practice (GCP) and the Declaration of Helsinki and the requirements of the Federal Regulations.

Please refer to:

International Conference on Harmonization and GCP:
http://www.fda.gov/oc/gcp/guidance.html

Declaration of Helsinki: http://www.fda.gov/oc/health/helsinki89.html

Code of Federal Regulations, Title 21:
http://www.accessdata.fda.gov/scripts/cdrh/cfdocs/cfcfr/CFRSearch.cfm]

8.3 Informed Consent

The informed consent documents must be signed and dated by the patient, or the patient's legally authorized representative, before his or her participation in the study. The case history for each patient shall document that informed consent was obtained prior to participation in the study. A copy of the informed consent documents must be provided to the patient or the patient's legally authorized representative.

8.4 Institutional Review Board

This protocol, the informed consent document, and relevant supporting information must be submitted to the IRB for review and must be approved before the study is initiated. In addition, any advertising materials must be approved by the IRB. The study will be conducted in accordance with applicable national and local health authority and IRB requirements.

The Sponsor-Investigator is responsible for keeping the IRB apprised of the progress of the study and of any changes made to the protocol as deemed appropriate, but in any case the IRB must be updated at least once a year. The Sponsor-Investigator must also keep the IRB informed of any significant adverse events.

The Sponsor-Investigator is required to promptly notify the IRB of all adverse drug reactions that are both serious and unexpected. This generally refers to serious adverse events that are not already identified in the Investigator Brochure and that are considered possibly or probably related to the study drug by the investigator. Some IRBs may have other specific adverse event requirements to which investigators are expected to adhere. Sponsor-Investigator must immediately forward to their IRB any written safety report or update provided by VentiRx Pharmaceuticals (e.g., IND safety report, Investigator Brochure, safety amendments and updates, etc.).

8.5 Retention of Records

U.S. FDA regulations (21 CFR §312.62[c]) require that records and documents pertaining to the conduct of this study and the distribution of investigational drug—including CRFs, consent forms, laboratory test results, and medication inventory records—must be retained by the Sponsor-Investigator for 2 years after marketing approval is received for VTX-2337, or for 2 years after all clinical and product development of VTX-2337 is discontinued and the applicable national and local health authorities are notified. The Sponsor-Investigator must notify VentiRx Pharmaceuticals prior to the destruction of any records relating to this study.

8.6 Drug Accountability

VentiRx Pharmaceuticals will provide the Sponsor-Investigator with adequate supplies of VTX-2337 for the study population and protocol requirements. Damaged supplies will be replaced. Drug supplies must be kept in an appropriate, secure area (e.g., locked

pharmacy) and stored in accordance with the conditions specified in this protocol and on the investigational drug labels.

Drug supplies are to be used only in accordance with this protocol. Investigational drug may be administered only to eligible subjects who are enrolled in the study, and the Sponsor-Investigator is accountable for all used and unused investigational drug. Used and partially used study drug will be destroyed according to the standard practice of the Investigational Pharmacy at the University of Washington / Seattle Cancer Care Alliance. Upon written notification, the Sponsor-Investigator will ship unused investigational drug according to instructions provided by VentiRx Pharmaceuticals, Inc. All material containing VTX-2337 will be treated and disposed of as hazardous waste in accordance with governing regulations.

Study drug accountability records should be maintained by the site in accordance with the regulations. A master drug log must be maintained of all VTX-2337 vials received, dispensed (including the lot number of the vials, the subject's ID number, the subject's initials, and the dates each vial is dispensed), and returned or destroyed. In addition, a subject-specific record of each vial administered, including the date and lot number of each vial, will be maintained with the case file for each subject. Any discrepancy in the drug distribution logs (master log and subject logs) must be explained in detail.

8.7 Premature Closure of the Study

This study may be prematurely terminated, if in the opinion of the Sponsor-Investigator or VentiRx Pharmaceuticals Inc, there is sufficient reasonable cause. Written notification documenting the reason for study termination will be provided to the Sponsor-Investigator or VentiRx Pharmaceuticals Inc by the terminating party.

Circumstances that may warrant termination include, but are not limited to:

- Determination of unexpected, significant, or unacceptable risk to patients.
- Failure to enter patients at an acceptable rate.
- Insufficient adherence to protocol requirements.
- Insufficient complete and/or evaluable data.
- Plans to modify, suspend or discontinue the development of the drug.

9.0 SUPPLEMENTS

9.1 References

1. Smith, E.M., et al., *Tobacco and alcohol use increases the risk of both HPV-associated and HPV-independent head and neck cancers.* Cancer Causes Control.
2. Carvalho, A.L., et al., *Trends in incidence and prognosis for head and neck cancer in the United States: a site-specific analysis of the SEER database.* Int J Cancer, 2005. **114**(5): p. 806-16.
3. Davies, L. and H.G. Welch, *Epidemiology of head and neck cancer in the United States.* Otolaryngol Head Neck Surg, 2006. **135**(3): p. 451-7.
4. Curado, M.P. and M. Hashibe, *Recent changes in the epidemiology of head and neck cancer.* Curr Opin Oncol, 2009. **21**(3): p. 194-200.
5. Hobbs, C.G., et al., *Human papillomavirus and head and neck cancer: a systematic review and meta-analysis.* Clin Otolaryngol, 2006. **31**(4): p. 259-66.
6. Dobrossy, L., *Epidemiology of head and neck cancer: magnitude of the problem.* Cancer Metastasis Rev, 2005. **24**(1): p. 9-17.
7. Specenier, P.M. and J.B. Vermorken, *Current concepts for the management of head and neck cancer: chemotherapy.* Oral Oncol, 2009. **45**(4-5): p. 409-15.
8. Rusthoven, K., et al., *Poor prognosis in patients with stage I and II oral tongue squamous cell carcinoma.* Cancer, 2008. **112**(2): p. 345-51.
9. Argiris, A., et al., *Head and neck cancer.* Lancet, 2008. **371**(9625): p. 1695-709.
10. Lango, M.N., *Multimodal treatment for head and neck cancer.* Surg Clin North Am, 2009. **89**(1): p. 43-52, viii.
11. Ferrari, D., et al., *A review on the treatment of relapsed/metastatic head and neck cancer.* Expert Opin Pharmacother, 2009. **10**(16): p. 2625-32.
12. Specenier, P.M. and J.B. Vermorken, *Recurrent head and neck cancer: current treatment and future prospects.* Expert Rev Anticancer Ther, 2008. **8**(3): p. 375-91.
13. Ferlito, A., et al., *Incidence and sites of distant metastases from head and neck cancer.* ORL J Otorhinolaryngol Relat Spec, 2001. **63**(4): p. 202-7.
14. Forastiere, A.A., *Chemotherapy in the treatment of locally advanced head and neck cancer.* J Surg Oncol, 2008. **97**(8): p. 701-7.
15. Boshoff, C. and M. Posner, *Targeting EGFR in head and neck cancer: a decade of progress.* Nat Clin Pract Oncol, 2009. **6**(3): p. 123.
16. Vermorken, J.B., et al., *Overview of the efficacy of cetuximab in recurrent and/or metastatic squamous cell carcinoma of the head and neck in patients who previously failed platinum-based therapies.* Cancer, 2008. **112**(12): p. 2710-9.
17. Vermorken, J.B., et al., *Open-label, uncontrolled, multicenter phase II study to evaluate the efficacy and toxicity of cetuximab as a single agent in patients with recurrent and/or metastatic squamous cell carcinoma of the head and neck who failed to respond to platinum-based therapy.* J Clin Oncol, 2007. **25**(16): p. 2171-7.
18. Rivera, F., et al., *Cetuximab in metastatic or recurrent head and neck cancer: the EXTREME trial.* Expert Rev Anticancer Ther, 2009. **9**(10): p. 1421-8.
19. Damiano, V., et al., *TLR9 agonist acts by different mechanisms synergizing with bevacizumab in sensitive and cetuximab-resistant colon cancer xenografts.* Proc Natl Acad Sci U S A, 2007. **104**(30): p. 12468-73.
20. Damiano, V., et al., *Novel toll-like receptor 9 agonist induces epidermal growth factor receptor (EGFR) inhibition and synergistic antitumor activity with EGFR inhibitors.* Clin Cancer Res, 2006. **12**(2): p. 577-83.

21. Pries, R., S. Wulff, and B. Wollenberg, *Toll-like receptor modulation in head and neck cancer.* Crit Rev Immunol, 2008. **28**(3): p. 201-13.
22. Rydberg, C., et al., *Toll-like receptor agonists induce inflammation and cell death in a model of head and neck squamous cell carcinomas.* Immunology, 2009. **128**(1 Suppl): p. e600-11.
23. Ventirx and Pharmaceuticals. *VentiRx Product Pipeline in Cancer Therapy: VTX-2337.* http://www.ventirx.com/product/cancer-vtx-2337.htm. 2010 [cited; Available from: http://www.ventirx.com/product/cancer-vtx-2337.htm.
24. Ventirx, *VentiRx VTX-2337 Investigator's Brochure*, in *unpublished*. 2010, version October 2010: Seattle, Washington.
25. Harari, P.M., D.L. Wheeler, and J.R. Grandis, *Molecular target approaches in head and neck cancer: epidermal growth factor receptor and beyond.* Semin Radiat Oncol, 2009. **19**(1): p. 63-8.
26. Astsaturov, I., R.B. Cohen, and P. Harari, *Targeting epidermal growth factor receptor signaling in the treatment of head and neck cancer.* Expert Rev Anticancer Ther, 2006. **6**(9): p. 1179-93.
27. Vincenzi, B., et al., *Cetuximab: from bench to bedside.* Curr Cancer Drug Targets. **10**(1): p. 80-95.
28. Vermorken, J.B., et al., *Platinum-based chemotherapy plus cetuximab in head and neck cancer.* N Engl J Med, 2008. **359**(11): p. 1116-27.
29. Baselga, J., et al., *Phase II multicenter study of the antiepidermal growth factor receptor monoclonal antibody cetuximab in combination with platinum-based chemotherapy in patients with platinum-refractory metastatic and/or recurrent squamous cell carcinoma of the head and neck.* J Clin Oncol, 2005. **23**(24): p. 5568-77.
30. Herbst, R.S., et al., *Phase II multicenter study of the epidermal growth factor receptor antibody cetuximab and cisplatin for recurrent and refractory squamous cell carcinoma of the head and neck.* J Clin Oncol, 2005. **23**(24): p. 5578-87.
31. Bernier, J., *Cetuximab in the treatment of head and neck cancer.* Expert Rev Anticancer Ther, 2006. **6**(11): p. 1539-52.
32. Burtness, B., *Cetuximab and cisplatin for chemotherapy-refractory squamous cell cancer of the head and neck.* J Clin Oncol, 2005. **23**(24): p. 5440-2.
33. Burtness, B., et al., *Phase III randomized trial of cisplatin plus placebo compared with cisplatin plus cetuximab in metastatic/recurrent head and neck cancer: an Eastern Cooperative Oncology Group study.* J Clin Oncol, 2005. **23**(34): p. 8646-54.
34. Burtness, B., *The role of cetuximab in the treatment of squamous cell cancer of the head and neck.* Expert Opin Biol Ther, 2005. **5**(8): p. 1085-93.
35. Vermorken, J.B. and P. Specenier, *Optimal treatment for recurrent/metastatic head and neck cancer.* Ann Oncol. **21 Suppl 7**: p. vii252-vii261.
36. Bonner, J.A., et al., *Radiotherapy plus cetuximab for locoregionally advanced head and neck cancer: 5-year survival data from a phase 3 randomised trial, and relation between cetuximab-induced rash and survival.* Lancet Oncol. **11**(1): p. 21-8.
37. Bonner, J.A., et al., *Radiotherapy plus cetuximab for squamous-cell carcinoma of the head and neck.* N Engl J Med, 2006. **354**(6): p. 567-78.
38. Kies, M.S. and P.M. Harari, *Cetuximab (Imclone/Merck/Bristol-Myers Squibb).* Curr Opin Investig Drugs, 2002. **3**(7): p. 1092-100.
39. Disis, M.L., et al., *Concurrent trastuzumab and HER2/neu-specific vaccination in patients with metastatic breast cancer.* J Clin Oncol, 2009. **27**(28): p. 4685-92.

9.2 Appendix A: Schedule of Protocol Activities and Assessments

	Protocol Activity	Screen Period	Cetuximab Lead-In					Cycle 1						Cycle 2				Cycle 3, 5, 7…	EOS	FU
		Week	-4	-3	-2	-1	-1	1	1	2	3	3	4	5	6	7	8			
		Day	-28	-21	-14	-7	-7 to -1*	1	2	8	15	16	22	29	36	42	50	1		+30
Baseline	Informed Consent [1]	X																		
Baseline	Medical History; Demographics	X																		
Baseline	BSA	X	(X)				X	(X)												
Baseline	Baseline Signs & Symptoms [2]	(X)	(X)				X													
Baseline	12-lead ECG [3]	X																		
Labs[4]	Hematology [5]	X	(X)	(X)	(X)	(X)	X	(X)		X	X		X	X	(X)	(X)	(X)	X	X	(X)
Labs[4]	Chemistry [6]	X	(X)	(X)	X	(X)	X	(X)		X	X		X	X	(X)	(X)	(X)	X	X	(X)
Labs[4]	Urinalysis [7]	X	(X)																	
Labs[4]	Pregnancy Test [8]	X																		
Rx	Cetuximab [9]		X	X	X	X		X		X	X		X	X	X	X	X	X		
Rx	VTX-2337 [10]							X		X	X			X	X	X		X		
Correlative Studies	Immune Monitoring [11]	X						2X	X		2X	X								
Correlative Studies	Flow Cytometry [12]	X						2X	X		2X	X								
Correlative Studies	ELISPOT [13]	X						X				X								
Correlative Studies	Pharmacogenetics [14]						X													
Assessments	Vital Signs; PE [15]	X	(X)	(X)	(X)	(X)	X	(X)			X		X	X	X	X	X	X	X	X
Assessments	Adverse Events [16]						X	(X)			X		X	X	X	X	X	X	X	X
Assessments	Tumor Imaging [17]	X[18]															X	X(Q8)	X	

* Patients will be assessed for cetuximab toxicities in this period (after the lead in dose(s) of cetuximab and prior to initiating combined treatment with VTX-2337 on Cycle 1, Day 1). If rash or severe toxicities (≥ Grade 3) due to cetuximab occur during lead-in period, patients will be taken off study, considered unevaluable and replaced.

(X) Optional; see endnote text
2X – peripheral blood draws will be done at two separate time points during this visit

[1] **Informed Consent**: Must be obtained prior to undergoing any study procedure and may occur up to 28 days prior to cetuximab run-in period.

[2] **Baseline Signs and Symptoms**: Patients will be asked about any signs or symptoms experienced within the past 14 days prior to Day 1. Baseline Assessment will be collected once for each patient, on Day -28 in lead-in period, and also in Assessment Period (within 7 days prior to Cycle 1 Day 1)

[3] **ECG**: 12-lead ECGs should be performed in the morning and time matched (±1 hour), and should be collected before or more than 30 minutes after needle sticks (e.g. phlebotomy and intravenous access procedures). It is preferable that the ECG machine used has a capacity to calculate the QTc interval. If the mean QTc interval is prolonged (>500 msec) or major abnormalities are present, then the ECGs should be reviewed by a Cardiologist at the clinical site for confirmation.

[4] **Laboratory Studies**: Clinical samples will be analyzed by local laboratories.

[5] **Hematology**: WBC with differential, hemoglobin, and platelet count. CBC may be drawn up to 2 days prior to cetuximab and / or VTX-2337 administration. If CBC is drawn during Screening within 7 days of cetuximab lead-in period, it is not necessary to repeat this test on Day -28 for cetuximab naïve subjects or Day -7 for subjects previously treated with cetuximab. Counts should be reviewed before each cycle, and therapy should be held if ANC <1500/μL and platelets <75,000.

[6] **Blood Chemistry**: Total bilirubin, AST, ALT, alkaline phosphatase, albumin, sodium, potassium, chloride, calcium, BUN, creatinine, glucose and magnesium. Blood chemistries may be drawn up to 2 days prior to cetuximab and/or VTX-2337 administration. If drawn during Screening, within 7 days of cetuximab lead-in period, it is not necessary to repeat these tests on Day -28 for cetuximab naïve subjects or day -7 for subjects previously treated with cetuximab

[7] **Urinalysis**: pH, specific gravity, protein, glucose, ketones, blood, leukocyte esterase, and nitrite.

[8] **Pregnancy Test (serum)**: Women of reproductive potential must be tested within 21 days starting study treatment.

[9] **Cetuximab**: Treatment with cetuximab starting on Day -28 (naïve subjects) or Day -7 (cetuximab veterans) with prophylactic antihistamine therapy prior to administration. Loading dose of 400 mg/m^2 given in lead-in period on Day -28 (naïve subjects) or a single cetuximab dose of 400 mg/m^2 (if greater than 3 weeks have elapsed since prior cetuxmab) or 250 mg/m^2 on Day -7 (subjects previously treated with cetuximab), then maintenance dosing weekly with 250 mg/m^2 IV. For cetuximab-naïve subjects, the lead-in period includes 3 weekly maintenance doses of cetuximab prior to the initiation of combination therapy. For subjects previously treated with cetuximab, the lead-in period consists only of the loading dose.

[10] **VTX-2337**: Treatment with VTX-2337 starts after assessment period on Week 1 (Cycle 1, Day 1). Dosing occurs for 3 consecutive weeks followed by a 1-week rest. VTX-2337 will be administered immediately following the cetuximab infusion.

[11] **Immune Monitoring**: (Performed by Rules-Based Medicine). Obtain Baseline samples for TruCulture and HuMAP analysis at Screening visit or prior to initial lead in dosing with cetuximab therapy.
On Cycle 1 Day 1, obtain sample prior to dosing with cetuximab (0hr), and then 8 hrs (± 15 minutes) and 24 hrs (± 1 hr; Day 2), after dosing with VTX-2337.
On Cycle 1 Day 15, obtain sample prior to dosing with cetuximab (0hr), and then 8 hrs (± 15 minutes) and 24 hrs (± 1 hr; Day 16), after dosing with VTX-2337.

[12] **Flow Cytometry**: (Performed by UW Tumor Vaccine Group.) Obtain Baseline sample at Screening visit or prior to initial lead in dosing with cetuximab.
On Cycle 1 Day 1, obtain sample prior to dosing with cetuximab (0hr), and then 8 hrs (± 15 minutes) and 24 hrs (± 1 hr; Day 2) after dosing with VTX-2337.
On Cycle 1 Day 15, obtain sample prior to dosing with cetuximab (0hr), and then 8 hrs (± 15 minutes) and 24 hrs (± 1 hr; Day 16) after dosing with VTX-2337.

[13] **ELISpot**: (Performed by UW Tumor Vaccine Group.) Obtain Baseline sample at Screening visit or prior to initial lead in dosing with cetuximab.
On Cycle 1 Day 1, obtain sample prior to dosing with cetuximab (0hr).
On Cycle 1 Day 16, obtain sample 24 hrs after Day 15 VTX-2337 dose is administered.

[14] **Pharmacogenetics**: (Performed by UW Tumor Vaccine Group.) Obtain saliva sample for genetic evaluation at any time prior to Cycle 1 Day 1.

[15] **Physical Examination** should include general assessment of organ systems in addition to specific evaluation of cancer related symptoms. This can be performed up to 1 day prior to cetuximab and/or VTX-2337 administration.

[16] **Adverse Events**: See §§5.2, 5.3, and 7.4 for Adverse Event details.

[17] **Tumor Imaging**: PET/CT, CT or MRI scan to be performed of the chest and other applicable sites to assess disease status at Screening. Thereafter, imaging should be performed following the completion of every 2 cycles of VTX-2337. The same imaging modality(ies) must be used for each patient throughout the study. Imaging should be done when disease progression is suspected; after 4 weeks or more following initial imaging demonstrating either PR or CR to confirm tumor response, and at the time of withdrawal from the study.

[18] **Brain imaging / Bone scan**: Brain CT or MRI should be performed at Screening if brain metastases are suspected. A bone scan should be performed at Screening if bone metastases are suspected.

9.3 Appendix B: Required Laboratory Tests

	Conventional Units	Conversion Factor	SI Units
**Hematology**			
Hemoglobin (Hgb)	g/dL	x 10	g/L
Platelet count (Plt)	$10^3/mm^3$	x 10^9	$10^{12}/L$
White blood count (WBC)	$10^3/mm^3$	x 10^6	$10^9/L$
White blood cell differential	%	x 0.01	fraction
**Chemistry**			
Total bilirubin	mg/dL	x 17.1	μmol/L
Alanine transaminase (ALT)	U/L	N/A	U/L
Aspartate transaminase (AST)	U/L	N/A	U/L
Alkaline phosphatase	U/L	N/A	U/L
Total protein	g/dL	x 10	g/L
Albumin	g/dL	x 10	g/L
Sodium	MEq/L	x 1.0	mmol/L
Potassium	MEq/L	x 1.0	mmol/L
Chloride	MEq/L	x 1.0	mmol/L
Calcium	mg/dL	x 0.25	mmol/L
Blood urea nitrogen (BUN)	mg/dL	x 0.357	mmol/L
Creatinine	mg/dL	x 88.4	μmol/L
Glucose	mg/dL	x 0.055	mmol/L
Magnesium	Meq/L	X10	Mmol/L

9.4 Appendix C: ECOG Performance Status

Grade	Description
0	Fully active, able to carry on all pre-disease performance without restriction
1	Restricted in physically strenuous activity but ambulatory and able to carry out work of a light or sedentary nature, e.g., light housework or office work
2	Ambulatory and capable of all self-care but unable to carry out any work activities. Up and about >50% of waking hours
3	Capable of only limited self-care, confined to a bed or chair >50% of waking hours
4	Completely disabled. Cannot carry on any self-care. Totally confined to bed or chair
5	Dead

9.5 Appendix D: RECIST Tumor Assessment Criteria

The determination of antitumor efficacy during this study will be based on objective tumor assessments made according to the RECIST system of unidimensional evaluation. A minor modification will be adopted to accommodate standard practice in use of spiral CT scan (i.e., reconstruction interval up to 8 mm). In the event spiral CT scan is used to assess tumors, minimum lesion size qualifying as measurable will be twice the reconstruction interval used and at least 10 mm.

Measurability of Tumor Lesions At baseline, individual tumor lesions will be categorized by the Investigator as either measurable or non-measurable by the RECIST criteria as described below.

- Measurable: Lesions that can be accurately measured in at least 1 dimension (longest diameter to be recorded) as ≥ 20 mm with conventional techniques or as ≥ 10 mm with spiral CT scan (depending on reconstruction interval). Clinical lesions will only be considered measurable when they are superficial (e.g., skin nodules, palpable lymph nodes).
- Non-Measurable: All other lesions, including small lesions and bone lesions, leptomeningeal disease, ascites, pleural or pericardial effusions, lymphangitis of the skin or lung, abdominal masses that are not confirmed and followed by imaging techniques, cystic lesions, previously irradiated lesions, and disease documented by indirect evidence only (e.g., by laboratory tests such as alkaline phosphatase).

NOTE: If measurable disease is restricted to a solitary lesion, its neoplastic nature should be confirmed by cytology/histology.

Recording Tumor Measurements

All measurable lesions up to a maximum of 5 lesions per organ and 10 lesions in total representative of all involved organs should be identified as target lesions and measured and recorded at baseline and at the stipulated intervals during treatment. Target lesions should be selected on the basis of their size (lesion with the longest diameters) and their suitability for accurate repetitive measurements (either by imaging techniques or clinically).

The longest diameter will be recorded for each target lesion. The sum of the longest diameter for all target lesions will be calculated and recorded as the baseline sum longest diameter to be used as reference to further characterize the objective tumor response of the measurable dimension of the disease during treatment. All measurements should be performed using a caliper or ruler.

All other lesions (or sites of disease) should be identified as non-target lesions and should also be recorded at baseline. Measurements are not required and these lesions should be followed as “present” or “absent.”

Techniques for Assessing Measurable Disease

The same method of assessment and the same technique should be used to characterize each identified and reported lesion at screening and during follow-up. Imaging-based evaluation is preferred to evaluation by clinical (physical) examination when both methods have been used to assess the antitumor effect of a treatment.

Definitions of Tumor Response

Target Lesions

- Complete response (CR) is defined as the disappearance of all target lesions.
- Partial response (PR) is defined as a 30% decrease in the sum of the longest dimensions of the target lesions taking as a reference the baseline sum longest dimensions.
- Progressive disease (PD) is defined as a 20% increase in the sum of the longest dimensions of the target lesions taking as a reference the smallest sum of the longest dimensions recorded since the treatment started, or the appearance of one or more new lesions.
- Stable disease (SD) is defined as neither sufficient shrinkage to qualify for PR nor sufficient increase to qualify for PD taking as a reference the smallest sum of the longest dimensions since the treatment started.

Non-Target Lesions

- Complete response (CR) is defined as the disappearance of all non-target lesions.
- Incomplete response (SD) is defined as a persistence of 1 non-target lesion.
- Progressive disease (PD) is defined as unequivocal progression of existing non-target lesions, or the appearance of new lesions.

The cytological confirmation of the neoplastic origin of any effusion that appears or worsens during treatment when the measurable tumor has met criteria for response or stable disease is mandatory to differentiate between response or stable disease and progressive disease.

Confirmation of Tumor Response

To be assigned a status of PR or CR, changes in tumor measurements in patients with responding tumors must be confirmed by repeat studies that should be performed ^{3}4 weeks after the criteria for response are first met.

Determination of Overall Response by the RECIST Criteria

When both target and non-target lesions are present, individual assessments will be recorded separately. The overall assessment of response will involve all parameters as depicted below.

Table 3: Response Evaluation Criteria in Solid Tumors

**Target Lesions[1]**	**Non-Target Lesions[2]**	**New Lesions[3]**	**Overall Response**
CR	CR	No	CR
CR	Incomplete response/SD	No	PR
PR	Non-PD	No	PR
SD	Non-PD	No	SD
PD	Any response	Yes or No	PD
Any response	PD	Yes or No	PD
Any response	Any response	Yes	PD

[1]Measurable lesions only.
[2]May include measurable lesions not followed as target lesions or non-measurable lesions.
[3]Measurable or non-measurable lesions.

The best overall response is the best response recorded from the start of the treatment until disease progression/recurrence (taking as reference for progressive disease the smallest measurements recorded since the treatment started). The patient's best response assignment will depend on the achievement of both measurement and confirmation criteria.

NOTE: Patients with a global deterioration of health status requiring discontinuation of treatment without objective evidence of disease progression at that time should be reported as "symptomatic deterioration." Every effort should be made to document the objective progression even after discontinuation of treatment.

In some circumstances, it may be difficult to distinguish residual disease from normal tissue. When the evaluation of complete response depends upon this determination, it is recommended that the residual lesion be investigated by fine needle aspirate or biopsy before confirming the complete response status.

9.6 Appendix E: NCI CTC AE

National Cancer Institute (NCI) Common Terminology Criteria for Adverse Events (CTCAE)

The NCI CTCAE (Version 4.0) will be used to assess adverse events for this protocol. The NCI CTCAE may be reviewed on-line at the following NCI website: http://ctep.cancer.gov/reporting/ctc.html

9.7 Appendix F: Correlative Immunologic Assays

VTX-2337 has been shown to induce biomarkers consistent with the activation of both innate and adaptive immunity in vitro and in vivo. Immunologic biomarkers proposed (see below) will assess markers representative of both arms of the immune system.

Additionally, genetic variants (single-nucleotide polymorphisms; SNPs) have been identified in both the TLR8 gene and Fc gamma-III gene which could potentially impact response to treatment with VTX-2337. Patient samples will undergo genotyping assays to allow genetic characterization of study participants.

9.7.1 Assessment of Baseline Immune Status

Baseline immune responses will be evaluated in vitro in all patients to explore the possibility that differences in immune responsiveness will correlate with clinical efficacy and/or safety. Blood samples will be obtained prior to the cetuximab run-in and evaluated using protein array technology (Rules Based Medicine, TruCulture™). The TruCulture closed-culture system provides a quantitative assessment of cytokine and chemokine responses to validated immunostimulatory agents.

Prior to initiating run-in with cetuximab, draw 1 mL of blood directly into the TruCulture tube using the provided kits. Place the culture tube immediately in a dry heat block at 37°C for 24–48 hours. After culture, follow the provided instructions to manually insert the valve into the culture tube to separate the supernatant from the cells. Using appropriate technique, transfer the plasma into an appropriately-labeled tube. **Freeze immediately at -70ºC until shipment.**

Frozen samples, including a sample manifest, should be stored until completion of study accrual. They should subsequently be shipped in an insulated container with dry ice to:

Rules-Based Medicine, Inc.
3300 Duval Road
Austin, TX 78759
Tel: 512.835.8026
Fax: 512.835.4687

Shipments must be marked for overnight delivery, and can be shipped on Monday through Thursday.

9.7.2 Assessment of Cytokine Response

Studies in non-human primates and in cancer patients have shown that specific cytokines, chemokines and other inflammatory markers are elevated in the peripheral blood of after administration of VTX-2337. Samples for immunologic monitoring (quantitative assessment for specific analytes by protein array technology; Rules Based Medicine HuMAP) will be obtained for each patient according to the schedule in Appendix A.

At the time points specified in Appendix A ("Immune Monitoring"), draw at least 3 mL of blood into a lavender-top K2 EDTA tube using standard venipuncture techniques. Invert the filled tube gently at least 8 to 10 times. [One complete inversion is to turn the filled tube upside-down and return it to the upright position.] Centrifuge the blood sample within 1 hour of collection time at 1500 x g for 15 minutes at 2ºC–8ºC until cells and plasma are well separated. Use a sterile pipette, transfer all the plasma into an appropriately-labeled tube. **Freeze immediately at -70ºC until shipment.**

Frozen samples, including a sample manifest, should be stored until completion of study accrual. They should subsequently be shipped in an insulated container with dry ice to:

Rules-Based Medicine, Inc.
3300 Duval Road
Austin, TX 78759
Tel: 512.835.8026
Fax: 512.835.4687

Shipments must be marked for overnight delivery, and can be shipped on Monday through Thursday.

9.7.3 Assessment of NK Cells

VTX-2337 has been shown to be a potent mediator of ADCC, effectively increasing target lysis in in-vitro culture. Assays will be performed (University of Washington Tumor Vaccine Group) to evaluate the activation and function of NK cells after administration of VTX-2337. Samples for assessment of NK cells via flow cytometry will be obtained for each patient according to the schedule in Appendix A.

At the time points specified in Appendix A ("Flow Cytometry"), draw at least 10 mL of blood into each of two green-top heparin tubes (20 mL total) using standard venipuncture techniques. Invert the filled tube gently at least 8 to10 times. [One complete inversion is to turn the filled tube upside-down and return it to the upright position.]

Tubes should be appropriately labeled and transported via same-day courier to:

University of Washington, Tumor Vaccine Group
Attn: Yushe Dang
815 Mercer Street, Room 219
Seattle, Washington 98109-8050
Telephone: (206) 616-8448

Upon receipt of samples at the TVG, CD56 cells will be stained and analyzed by flow cytometry for surface markers: CD69 (activation) and intracellular stains: IFN-gamma and CD107 (function-cytokine secretion and degranulation). Separate reactions will be performed for IFN-gamma and CD107 (i.e. each staining will include 2 surfaces and 1 intracellular stain).

9.7.4 IFN-gamma ELISPOT:

VTX-2337 has been shown to activate IL-12 secreting dendritic cell populations in human PBMC. Consequently, administration of VTX-2337 may stimulate adaptive immune responses. Several immunogenic proteins—including EGFR and CD105 —have been identified as biologically relevant antigens for head and neck cancer. In multivariate analyses, these proteins have been found to be indicators of poor clinical outcome when overexpressed in head and neck tumors. Moreover, these proteins have been shown to be human tumor antigens by the UW Tumor Vaccine Group and others. Antigen-specific IFN-gamma secretion has been shown to be correlated to beneficial clinical outcome after immunotherapy. Preliminary information as to the ability to stimulate such responses after administration of VTX-2337 will be collected. Samples for assessment by ELISpot will be obtained for each patient according to the schedule in Appendix A.

At the time points specified in Appendix A ("ELISPOT"), draw at least 10 mL of blood into <u>each</u> of <u>eight</u> green-top heparin tubes (80 mL total) using standard venipuncture techniques. Invert the filled tube gently at least 8 to 10 times. [One complete inversion is to turn the filled tube upside-down and return it to the upright position.]

Tubes should be appropriately labeled and transported via same-day courier to:

University of Washington, Tumor Vaccine Group
Attn: Yushe Dang
815 Mercer Street, Room 219
Seattle, Washington 98109-8050
Telephone: (206) 616-8448

Upon receipt of samples at the TVG, PBMC will be purified from peripheral blood and plated at $1x10^5$/well in quadruplicate for each antigen. A 10-day ELISpot will be performed in accordance to the methods described by Disis et. al.[39] All samples for individual patients will be cryopreserved then reconstituted and analyzed at the same time at the end of study to correct for variation. Antigens to be tested include:

- No antigens (media only)
- HIV peptide (negative antigen control)
- recombinant proteins for EGFR, HIF-1a, CD105 and Fascin-1 at 1 ug/ml
- CEF peptide pool (positive antigen control)
- PHA

9.7.5 Genetic Analysis

Functional genetic variants (single-nucleotide polymorphisms; SNPs) have been identified in both the TLR8 gene (the A1G allele) and Fcγ Receptor IIIA gene (158 F allele) in humans. Functional SNPs could potentially impact response to treatment with VTX-2337. To assess whether patients with SNPs have altered biological and/or clinical responses to VTX-2337 as compared to patients without such polymorphisms, a sample will be obtained from all patients enrolled in the study for pharmacogenomic analysis.

A saliva sample will be collected from each eligible subject prior to the Cycle 1 Day 1 dose of VTX-2337. Samples should be appropriately labeled and transported to:

University of Washington, Tumor Vaccine Group
Attention: Yi Yang
815 Mercer Street, Room 219
Seattle, Washington 98109
Telephone: (206) 616-8448

Upon receipt of samples at the TVG, pharmacogenomic analysis will be performed to characterize SNPs in the TLR8 and FcγR IIIA genes by conventional methods.

9.8 Appendix G: Guidelines for Treatment of Ocular Toxicities

<u>Guidelines for Treatment of Anterior Inflammation</u>

- homatropine ophthalmic, 5%: one drop in the affected eye every 12 hours
- prednisolone ophthalmic, 1%: 1 drop in the affected eye every hour while awake (≥ 12 doses per day)
- consider topical medications to reduce intraocular pressure as indicated

<u>Guidelines for treatment of posterior inflammation:</u>

- homatropine ophthalmic, 5%: one drop in the affected eye every 12 hours
- prednisolone ophthalmic, 1%: 1 drop in the affected eye every hour while awake (≥12 doses per day)
- 60-80 mg, or approximately 1 mg/kg, of prednisone or prednisolone equivalent by mouth per day
- Consider intraocular steroid injections as indicated
